# Quantification by qPCR of Pathobionts in Chronic Periodontitis: Development of Predictive Models of Disease Severity at Site-Specific Level

**DOI:** 10.3389/fmicb.2017.01443

**Published:** 2017-08-09

**Authors:** Inmaculada Tomás, Alba Regueira-Iglesias, Maria López, Nora Arias-Bujanda, Lourdes Novoa, Carlos Balsa-Castro, Maria Tomás

**Affiliations:** ^1^Oral Sciences Research Group, Department of Surgery and Medical Surgical Specialties, School of Medicine and Dentistry, Universidade de Santiago de Compostela, Health Research Institute of Santiago (IDIS) Santiago de Compostela, Spain; ^2^Department of Microbiology, Complejo Hospitalario Universitario A Coruña-Instituto de Investigación Biomédica de A Coruña A Coruña, Spain

**Keywords:** chronic periodontitis, multivariate modeling techniques, paired design, periopathogens, predictive ability, qPCR, site-specific, subgingival plaque

## Abstract

Currently, there is little evidence available on the development of predictive models for the diagnosis or prognosis of chronic periodontitis based on the qPCR quantification of subgingival pathobionts. Our objectives were to: (1) analyze and internally validate pathobiont-based models that could be used to distinguish different periodontal conditions at site-specific level within the same patient with chronic periodontitis; (2) develop nomograms derived from predictive models. Subgingival plaque samples were obtained from control and periodontal sites (probing pocket depth and clinical attachment loss <4 mm and >4 mm, respectively) from 40 patients with moderate-severe generalized chronic periodontitis. The samples were analyzed by qPCR using TaqMan probes and specific primers to determine the concentrations of *Actinobacillus actinomycetemcomitans (Aa)*, *Fusobacterium nucleatum (Fn)*, *Parvimonas micra (Pm)*, *Porphyromonas gingivalis (Pg)*, *Prevotella intermedia (Pi)*, *Tannerella forsythia (Tf)*, and *Treponema denticola (Td)*. The pathobiont-based models were obtained using multivariate binary logistic regression. The best models were selected according to specified criteria. The discrimination was assessed using receiver operating characteristic curves and numerous classification measures were thus obtained. The nomograms were built based on the best predictive models. Eight bacterial cluster-based models showed an area under the curve (AUC) ≥0.760 and a sensitivity and specificity ≥75.0%. The *PiTfFn* cluster showed an AUC of 0.773 (sensitivity and specificity = 75.0%). When *Pm* and *AaPm* were incorporated in the *TdPiTfFn* cluster, we detected the two best predictive models with an AUC of 0.788 and 0.789, respectively (sensitivity and specificity = 77.5%). The *TdPiTfAa* cluster had an AUC of 0.785 (sensitivity and specificity = 75.0%). When *Pm* was incorporated in this cluster, a new predictive model appeared with better AUC and specificity values (0.787 and 80.0%, respectively). Distinct clusters formed by species with different etiopathogenic role (belonging to different Socransky’s complexes) had a good predictive accuracy for distinguishing a site with periodontal destruction in a periodontal patient. The predictive clusters with the lowest number of bacteria were *PiTfFn* and *TdPiTfAa*, while *TdPiTfAaFnPm* had the highest number. In all the developed nomograms, high concentrations of these clusters were associated with an increased probability of having a periodontal site in a patient with chronic periodontitis.

## Introduction

Periodontal diseases are among the most common conditions affecting human beings (Dentino et al., 2013). In 2010, severe periodontitis was estimated to be the sixth most prevalent disease globally, affecting 743 million people worldwide and with an age-standardized incidence of 701 cases per 100,000 person-years (Kassebaum et al., 2014). Periodontitis is an inflammatory condition of the gingivae. It causes the destruction of the ligament and alveolar bone supporting the teeth, resulting in oral malodour and tooth loss (Dentino et al., 2013). In addition to its impact on the oral health status and quality of life of patients (Shanbhag et al., 2012; Al-Harthi et al., 2013), periodontitis is currently being connected bidirectionally to the pathogenesis of various systemic diseases and conditions such as diabetes (Chapple et al., 2013), coronary heart disease (Tonetti et al., 2013), rheumatoid arthritis (de Pablo et al., 2009), respiratory diseases (Bansal et al., 2013), and dementia (Abbayya et al., 2015).

Although traditional clinical measures are informative for evaluating the severity of periodontitis and the response to therapy (Korte and Kinney, 2016), these clinical criteria are only partially able to determine current disease activity or the future risk of structure loss (Giannobile et al., 2009; Zhang et al., 2009). As a result, one of the major challenges in the field of periodontology is to determine biomarkers for screening and predicting the early onset of periodontitis or evaluating disease activity and the efficacy of therapy (diagnostic or prognostic tests) (Zhang et al., 2009; Buduneli and Kinane, 2011).

Periodontal diseases are multifactorial in origin; their initiation and progression require the involvement of several factors, including bacteria that contribute to the formation of a polymicrobial biofilm at the subgingival level. Consequently, periodontal microbiology has been an area of intense research for decades (Teles et al., 2013).

In order to overcome the well-known limitations of bacterial cultures, a series of molecular techniques were developed, including DNA hybridization and the polymerase chain reaction (PCR) (Socransky et al., 1998; Loomer, 2004). Socransky et al. (1998) used genomic hybridization techniques to identify three species that were strongly associated with the clinical parameters of chronic periodontitis: *Treponema denticola*, *Porphyromonas gingivalis*, and *Tannerella forsythia*. These species together constituted the so-called ‘Red Complex.’ This complex was related to other bacteria such as *Fusobacterium nucleatum*, *Prevotella intermedia*, *Prevotella nigrescens*, *Peptostreptococcus micros (Parvimonas micra)*, *Campylobacter rectus*, *Campylobacter showae*, *Campylobacter gracilis*, *Eubacterium nodatum*, *Streptococcus constellatus*, and *Fusobacterium periodonticum*. All of these species formed the ‘Orange Complex.’

Quantitative PCR, qPCR or real-time PCR are variants of the conventional PCR technique that is used to amplify and simultaneously quantify the amplification product obtained from a sample (Sakamoto et al., 2005). There are several studies in the scientific literature in which the diagnostic accuracy of the qPCR technique in samples from healthy subjects and periodontitis patients was assessed using the conventional bacterial culture as a reference (Boutaga et al., 2005, 2006, 2007; Jervoe-Storm et al., 2005; Atieh, 2008; Kotsilkov et al., 2015). The conclusion that can be drawn from these investigations is that the qPCR is a sensitive and specific method which has a high positive predictive value (PPV) when it comes to the detection of target bacteria (Boutaga et al., 2005, 2006, 2007; Jervoe-Storm et al., 2005; Atieh, 2008; Kotsilkov et al., 2015). In fact, Atieh (2008) performed a meta-analysis in 2008 in which it was demonstrated that this molecular technique is associated with a high diagnostic accuracy in detecting (specifically) *Aggregatibacter actinomycetemcomitans* and *P. gingivalis* compared to conventional cultures.

Until now, research on periodontal microbiology using qPCR has focused on the study of periopathogens such as: *A. actinomycetemcomitans*, *P. gingivalis*, *T. forsythia*, *P. intermedia*, *T. denticola*, *F. nucleatum*, and *P. micra* (Braga et al., 2010; Saygun et al., 2011; Decat et al., 2012; Polonyi et al., 2013; Gatto et al., 2014; Kinney et al., 2014; Al-hebshi et al., 2015; Kotsilkov et al., 2015; Milne et al., 2015; Scapoli et al., 2015; Torrungruang et al., 2015; Golynska et al., 2016). In most of these q-PCR studies, the authors applied a ‘simple analytical approach’ consisting of univariate comparisons of the periopathogen levels quantified in different periodontal conditions (Braga et al., 2010; Polonyi et al., 2013; Kotsilkov et al., 2015; Milne et al., 2015; Scapoli et al., 2015; Golynska et al., 2016). As a result, there are very few studies based on a multivariate modeling approach (Saygun et al., 2011; Gatto et al., 2014; Kinney et al., 2014; Al-hebshi et al., 2015; Torrungruang et al., 2015).

Consequently, when assuming that chronic periodontitis is orchestrated by a bacterial consortium rather than by a single pathogen (Teles et al., 2013), further evidence is required on the development of predictive models for diagnosing periodontitis or its prognosis based on the qPCR quantification of subgingival pathobionts using appropriate multivariate analytical techniques. Accordingly, the objectives of the present cross-sectional study (paired design) were to:

(1) Compare the detection frequencies and levels of seven well-known pathobionts detected in subgingival sites with different periodontal conditions (control site vs. periodontal site) within the same patient with chronic periodontitis.
(2) Obtain pathobiont-based predictive models that could be used to distinguish different periodontal conditions at site-specific level within the same periodontal patient.
(3) Develop nomograms derived from pathobiont-based predictive models.

## Materials and Methods

### Selection of Study Group and Clinical Examination

A total of 40 subjects affected by moderate to severe generalized chronic periodontitis were recruited among consecutive patients who were referred between 2014 and 2016 to the Periodontology and Patients with Special Needs units at the School of Medicine and Dentistry (Universidade de Santiago de Compostela, Spain) for an assessment of their oral health status. Patients were selected if they fulfilled the following inclusion criteria: (1) age 30 to 65 years; (2) good general health, no pregnancy or breastfeeding; (3) no intake of systemic antimicrobials during the previous 6 months; (4) no intake of antiinflammatory medication in the previous 4 months; (5) no routine use of oral antiseptics; (6) no presence of implants or orthodontic appliances; (7) no previous periodontal treatment; and (8) the presence of at least 18 natural teeth.

**FIGURE 1:**
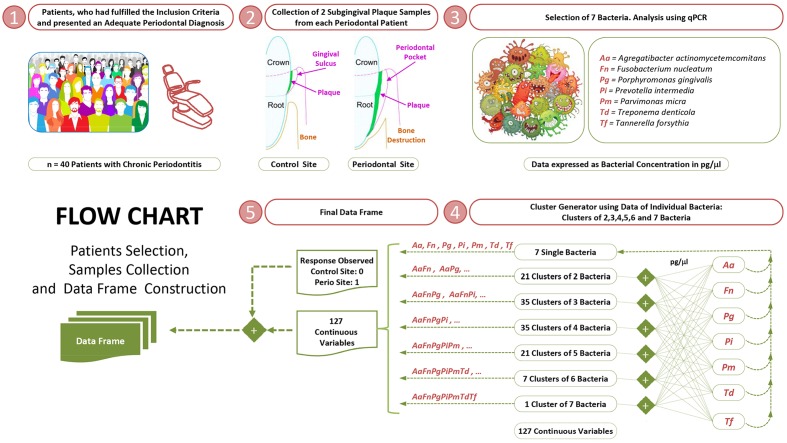
Selection of study groups, collection of subgingival samples and construction of dataframe.

Two experienced dentists and previously calibrated performed all the periodontal diagnoses. The probing pocket depth (PPD) and clinical attachment loss (CAL) (=PPD+ gingival recession) were recorded throughout the mouth on six sites per tooth (excluding third molars) using a PCP-UNC 15 probe. Bleeding on probing (BOP) and bacterial plaque levels (BPL) were recorded for the full mouth on a binary scale (presence/absence) on six sites per tooth. Standardized radiographs of all teeth were obtained to assess the alveolar bone status. Patients were diagnosed as suffering from moderate to severe generalized chronic periodontitis based on the previously established criteria (Armitage, 1999; Page and Eke, 2007). Smoking status was also recorded (**Figure 1**).

Patients who agreed to participate in the study provided their written informed consent. The study protocol was approved by the Ethics Committee of Clinical Investigations of Galicia (registration number 2015/006). The ‘transparent reporting of a multivariable prediction model for individual prognosis or diagnosis’ (TRIPOD) guidelines were considered (Moons et al., 2015).

### Collection of Subgingival Plaque Samples

Control and periodontal sites were defined within the same periodontal patient as follows: (1) the control sites had a PPD and CAL <4 mm and no radiographic evidence of alveolar bone loss; and (2) the periodontal sites had a PPD and CAL >4 mm and radiographic evidence of bone loss (**Figure 1**).

All the subgingival plaque samples were collected in the afternoon, approximately 5–7 h after toothbrushing. The samples were obtained from both a control site and a periodontal site (two or three adjacent locations in each site) in each periodontal patient by inserting a total of five sterile endodontic paper points into each location (size 30) for 10 s following isolation and supragingival plaque removal. The samples were placed in 1.5 ml microcentrifuge tubes with 300 μl of a phosphate buffer and frozen at -80°C until further analysis.

### DNA Extraction and qPCR

Two investigators blinded to the clinical data performed the bacterial analyses by qPCR. The bacteria were separated from the paper points before the DNA extraction by washing with 500 μL of a phosphate buffer solution and vortexing. DNA extraction from the samples was performed in the MagNA Pure Compact Instrument (Roche, Germany) following the manufacturer’s instructions. We used it in combination with the MagNA Pure Bacteria Lysis Buffer (Roche) and the DNA Bacteria purification protocol (Roche) to work with the bacterial DNA isolated from subgingival plaque samples.

The levels of seven pathobionts were quantified: *A. actinomycetemcomitans (Aa); F. nucleatum (Fn); P. micra (Pm); P. gingivalis (Pg); P. intermedia (Pi); T. forsythensis (Tf);* and *T. denticola (Td).* Real-time PCR (LightCycler^®^480 system, Roche) was performed using TaqMan probes (Universal Probe Library, Roche) and primers were designed to amplify specific target genes from each bacterium (**Table 1**) (Fernández-Pinero et al., 2013; Fraczyk et al., 2016). The specificity of the probes was analyzed among all the bacteria from this study. We worked with uracil-*N*-glycosylase, Cod-UNG (ArcticZymes, Norway) (Bou et al., 2014) to remove contaminating amplicons from the previous qPCR. To confirm this, we used the water from the reaction of the qPCR as a negative control (Champlot et al., 2010).

**Table 1:** DNA regions amplified, the targeted gene name, TaqMan probes and primers used in the present study for the detection of the seven bacteria analyzed.

Bacteria	DNA region amplified (bp)	Genes (Genbank; Acc. number)	UPL probes (Number)	Primers (5′–3′)	Controls
*Aa*	CAATACTACGGTGGTGCAGTATC TGCACCGTTGTTCTCCAGCATTA TGGGTTACGCATTACGCGCCAAC AATAT	*ftsI* (gb|CP001733.1| : 702960–704783)	CTCCAGCA (number 67)	5′-CAATACTACGGTGGTGCAGTATCT-3′ 5′-ATATTGTTGGCGCGTAATGC-3′	ATCC^®^ 700685^TM^
*Fn*	TCCCAGCAAATGTTGGAAGAAT TGAATATGCTGAAGAAGAAGAT GAAGACTATGACGAATTTGATG ATGAA	*ftsX* (gb|AE009951.2| : 891544–892470)	AGAAGAAGA (number 143)	5′- TCCCAGCAAATGTTGGAAG-3′ 5′-TTCATCATCAAATTCGTCATAGTCT-3′	ATCC^®^ 25611^TM^
*Pm*	AGAATCAATTTCTCAAGGTGCA GAAGCTGAAGGAAAATACATCA GACAGTCTGGTGGTAGTGGACA ATATGGACATTG	*fusA* (gb|CP009761.1| : 310535–312610)	GCTGAAGG (number 161)	5′-AGAATCAATTTCTCAAGGTGCAG-3′ 5′-CAATGTCCATATTGTCCACTACCA-3′	ATCC^®^ 33270^TM^
*Pg*	ATAGTAGCGTGTCCGGCTTCGTG GATGGCGATGCTGCGACGCTCCT CCTCTGTGGTGATTTTGTTCTTTT TCTCCAATCCGCCTACGAT	*ftsH* (gi|188593544| : 54171–56192)	CTCCTCTG (number 82)	5′-ATAGTAGCGTGTCCGGCTTC-3′ 5′-ATCGTAGGCGGATTGGAGA-3′	ATCC^®^ 33277^TM^
*Pi*	TGGTATCAAAATCAGCAAGGAA ACAACACCAGAGATTTATAAGC TTGTGCTGAATATGCGCGAAGACG	*piACP* (gb|CP019300.1| : 638473–639264)	ACCAGAGA (number 126)	5′- TGGTATCAAAATCAGCAAGGAA-3′ 5′- CGTCTTCGCGCATATTCAG-3′	ATCC^®^ 25611^TM^
*Tf*	AAACATCGTGGATACCCTCCTTA TACATATGTGTGACAGCGTTTCC GCCGCCACCACCGACACCGACC ACTTTGAT	*ftsZ* (gb|CP003191.1| : 422801–424174)	CCGCCGCC (number 70)	5′-AAACATCGTGGATACCCTCCT-3′ 5′-ATCAAAGTGGTCGGTGTCG-3′	ATCC^®^ 43037^TM^
*Td*	GCAGATATACAGGTAGACATAG GAAGCGCAGCTCCTGAAGAATC CAAAAAAAGCATTATAGAATCC CTTGTCGTAATAGAAGATATAA ACACTGAACAGGACTTAGAAGAGC	*ftsK* (gb|AE017226.1| : 204110–206650)	AGCTCCTG (number 128)	5′-CAGATATACAGGTAGACATAGGAAGC-3′ 5′-GCTCTTCTAAGTCCTGTTCAGTGTT-3′	VPI 4355

*Aa, A. actinomycetemcomitans; Fn, F. nucleatum; Pm, P. micra; Pg, P. gingivalis; Pi, P. intermedia; Tf, T. forsythia; Td, T. denticola.*

Absolute quantification of the DNA was performed using the standard curve method. A standard dilution series of known amounts of genomic DNA (from 100 pg to 1 fg) were assessed in the same assay. Cp-values obtained from these standards were used to generate a standard curve from which the amount of DNA in the unknown samples was calculated. Each standard contained the same target region that was amplified in the sample. Strains from each microorganism, obtained from the ATCC Bacteriology Collection, were used both to generate the standard curves and as positive controls. A regression analysis was performed to obtain the equation used to interpolate the Cp-values from healthy and diseased sites and thus quantify the corresponding concentrations of genomic DNA of each pathobiont in each sample (pg/μl). The value of *R*^2^ was used as a measure of the goodness-of-fit of the regression analysis.

### Statistical Analysis

The unit of analysis in the study was the sampled subgingival site. The ‘*a priori*’ sample size calculation was performed using the program G^∗^Power 3.1.5 (Faul et al., 2007). The following statistical criteria were established: (1) an effect size of 0.5 for bacterial concentrations between the control and periodontal sites; (2) an alpha error of 0.05; and (3) a statistical power of 90%. A sample size of 36 subjects was required on the basis of these criteria and the application of the Wilcoxon test to evaluate the differential bacterial concentrations.

The independent variables were: (1) the quantitative levels of the species individually (7 variables); and (2) due to its biological significance, the quantitative levels of all possible bacterial clusters (120 variables) (**Figure 1**) (**Supplementary Data Sheet S1**). The statistical analyses were performed using the R software (R Core Team, 2016).

#### Univariate Analysis of the Detection Frequencies and Levels of Pathobionts in Relation to the Severity of Chronic Periodontitis: Control Sites vs. Periodontal Sites

Contingency tables were used and the McNemar test was applied to compare the detection frequencies between the control and periodontal sites. The Shapiro–Wilk test was performed to analyze the distribution of the quantitative variables. As the clinical parameters associated with chronic periodontitis, as well as the subgingival concentrations of the pathobionts, showed a non-normal distribution, the contrast between the two types of subgingival site (control vs. periodontal) was determined using the Wilcoxon test.

The Student’s *t*-test or Mann–Whitney *U* test for quantitative variables (according to the type of distribution of variables) and the Fisher’s exact test for categorical variables were used to compare the clinical characteristics between smokers and non-smokers. The influence of smoking (smokers vs. non-smokers) in the detection frequencies and bacterial concentrations for each type of subgingival site were studied using the Fisher’s exact test and the Mann–Whitney *U* test, respectively.

The Benjamini–Hochberg correction was applied to control the false discovery rate (FDR) (Benjamini and Hochberg, 1995) in the comparative analysis, establishing a Q parameter of 0.05, which corresponds to a FDR < 5%. A significance level of *P* < 0.05 was applied.

Correlations of bacterial levels with clinical parameters (BOP, PPD, and CAL) were assessed using Spearman’s correlation coefficients.

#### Multivariate Predictive Modeling of Chronic Periodontitis at Site-Specific Level Based on Subgingival Pathobiont Levels: Selection of Models

Spearman correlations between pathobionts were calculated and used as an orientation for model building, in order to prevent redundancies and possible collinearity between bacteria with similar biological effects. Pathobiont-based models were selected for their biological significance, their capacity to predict chronic periodontitis at site-specific level and their statistical validity.

Models were constructed by initially selecting one bacterium or bacterial cluster as predictor variable. In order to test whether the predictive ability of bacterial clusters can be increased by incorporating others, two-variable models combining different bacterial clusters were analyzed. A methodological advantage of the matched case-control sample approach within the same periodontal patient used in this study is that the subject-associated variables (such as age, gender, and smoking status) are the same for the control and periodontal sites and so do not need to be incorporated in the predictive models.

The statistical criteria applied for model selection were:

(1) The capacity of each pathobiont-based model to discriminate the severity of chronic periodontitis at site-specific level, that was assessed with the Epi package and using the receiver operating characteristic (ROC) curve (Carstensen et al., 2017). The models with an area under the curve (AUC) value ≥0.76 were selected, as these are typically considered to be acceptable predictive models (Hosmer et al., 2013). The calculation of the AUC values and their corresponding 95% confidence intervals (CIs) by bootstrapping was performed using the pROC package (Robin et al., 2011).
(2) The capacity of each pathobiont-based model to classify the severity of chronic periodontitis at site-specific level, that was assessed with the pROC package and bootstrapping. Numerous classification measures such as accuracy (ACC), sensitivity, specificity, the PPV, and the negative predictive value (NPV) were obtained by setting an optimal threshold, as well as their corresponding 95% CIs (Robin et al., 2011). The best cut-off value for each model was determined so that the percentage of correct predictions was the maximum. The models with sensitivity and specificity values ≥75% were selected.
As a single indicator of diagnostic performance, the diagnostic odds ratio (DOR) was calculated as the ratio of the odds of positivity in the diseased patients relative to the odds of positivity in those with no disease (Glas et al., 2003). The value of the DOR ranges from 0 to infinity, with higher values indicating a better discriminatory test performance (higher accuracy). A DOR of 1.0 indicates that a test does not discriminate between subjects with the disease and those without it. DOR values <1.0 suggest improper test interpretation (a proportion of negative test results in the group with disease) (Glas et al., 2003).
(3) In models with at least two independent variables, the variance inflation factor (VIF; for models which only include continuous variables) was <2.28. This value allowed us to assume that the model did not present evidence of multicollinearity between the independent variables (O’brien, 2007).
(4) The Hosmer–Lemeshow test (a calibration measure) was not significant (*P* > 0.05) when using the Resource Selection package (Lele et al., 2017).

Any model that did not fulfill any of these inclusion criteria was discarded for the posterior analysis.

#### Multivariate Predictive Modeling of Chronic Periodontitis at Site-Specific Level Based on Subgingival Pathobiont Levels: Validation of Selected Models, and the Development of Nomograms

Bootstrap methods were used to test for possible overfitting by determining optimism values on the discrimination and classification measures. The bootstrap analysis was replicated on 1000 different samples of the same sample size drawn with replacements from the original sample. Optimism, which is a measurement of internal model validation that refers to the absolute magnitude of bias, equals the difference between respective statistics of the bootstrap sample (bootstrap performance) and the bootstrap model in the original sample (test performance) (Efron and Tibshirani, 1994; Steyerberg et al., 2001b). Bias-corrected (bc) AUC values and all the classification measures (bc-ACC, bc-sensitivity, bc-specificity, bc-PPV, and bc-NPV) were calculated as their corresponding apparent measures derived from the entire original sample minus optimism (Efron and Tibshirani, 1994; Steyerberg et al., 2001b). In terms of the bc-DOR, these ratios were calculated from the values of bc-sensitivity and bc-specificity.

The nomograms were built based on the selected models using the RMS package (Harrel, 2016). A nomogram maps the predicted probabilities into points on a scale from zero to 100 in a user-friendly graphical interface. The total points accumulated by the various covariates correspond to the predicted probability of having a subgingival site with periodontal destruction in a periodontal patient (Iasonos et al., 2008).

**FIGURE 2:**
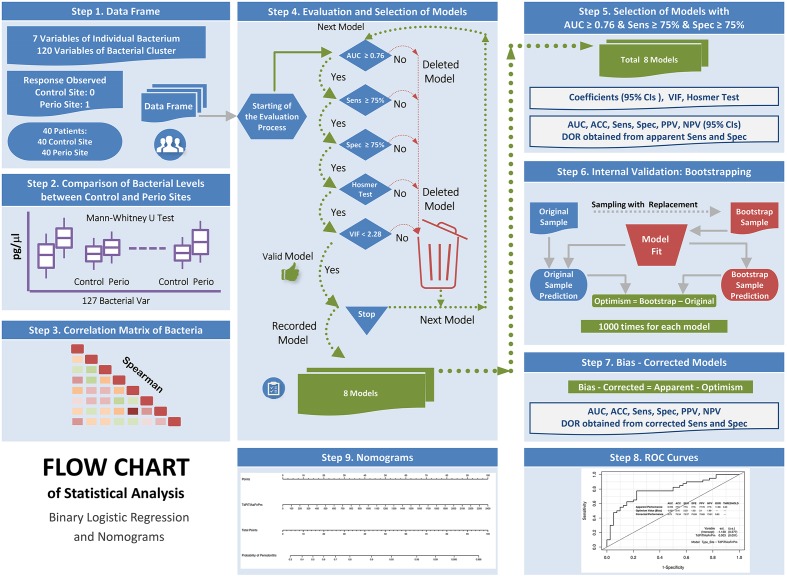
Flow chart of the statistical analysis: binary logistic regression and diagnostic nomograms.

**Figure 2** shows the flow chart of the statistical analysis: binary logistic regression and diagnostic nomograms.

## Results

### Characteristics of the Study Group

The study group consisted of 40 individuals with a diagnosis of moderate-severe generalized chronic periodontitis and an average age of 51.35 years. Of these patients, 18 were male and 22 female. With regard to the comparative analysis of clinical parameters between the control and periodontal sites, the latter showed significantly higher BOP, PPD, and CAL values (**Table 2**).

**Table 2:** Demographic and clinical characteristics of the patients with chronic periodontitis included in the present study, as well as the clinical characteristics of the sampled subgingival sites (control site vs. periodontal site).

Clinical characteristics of patients (*n* = 40)			
Age, years; Mean ± SD		51.35 ± 8.31	–
Gender; No. and %	Male	18	45.00%
	Female	22	55.00%
Smoking habit^1^; No. and %	Never	19	47.50%
	Current	21	52.50%
Teeth present, No.; Mean ± SD		25.80 ± 3.93	–
BPL full mouth, % sites; Mean ± SD		–	55.43% ± 29.25%
BOP full mouth, % sites; Mean ± SD		–	47.15% ± 21.32%
PPD full mouth, mm; Mean ± SD		3.65 ± 0.63	–
CAL full mouth, mm; Mean ± SD		4.55 ± 1.08	–
PPD full mouth, % sites	<4 mm	–	55.62%
	4–6 mm	–	39.76%
	>6 mm	–	4.62%
CAL full mouth, % sites	<4 mm	–	38.43%
	4–6 mm	–	46.49%
	>6 mm	–	15.08%

**Clinical characteristics of sampled subgingival sites (*n* = 80)**		**Control site (*n* = 40)**	**Periodontal site (*n* = 40)**

BOP, % sites; Mean ± SD		44.17% ± 41.61%^∗^	70.83% ± 34.75%^∗^
BOP, % sites; Median (IQR)		50% (100.00%)^∗^	83.34% (66.67%)^∗^
PPD, mm; Mean ± SD		2.68 ± 0.32^∗∗^	5.90 ± 1.49^∗∗^
PPD, mm; Median (IQR)		2.67 (0.50)^∗∗^	5.50 (1.00)^∗∗^
CAL, mm; Mean ± SD		2.96 ± 0.61^∗∗^	6.28 ± 1.79^∗∗^
CAL, mm; Median (IQR)		2.84 (1.00)^∗∗^	5.84 (1.75)^∗∗^

^1^*In terms of smoking habit, a patient was considered to be: a current smoker if he/she currently smoked and had a smoking history of at least 8 years; a non-smoker if he/she had never smoked or had stopped smoking at least 5 years ago; and a former smoker if he/she had stopped smoking less than 5 years ago*.

*BPL, bacterial plaque levels; BOP, bleeding on probing; PPD, probing pocket depth; CAL, clinical attachment loss; SD, standard deviation; IQR, interquartile range;*

*^∗^*P* = 0.001, ^∗∗^*P* < 0.001*.

In relation to their smoking habits, 19 patients were non-smokers and 21 current smokers, and both patient groups showed similar age and gender characteristics, as well as similar values in terms of the periodontal indices recorded in the full mouth and the sampled subgingival sites.

### Detection Frequencies and Levels of Pathobionts in the Chronic Periodontitis: Control Sites vs. Periodontal Sites

The detection frequencies of the seven species evaluated were similar between the control and periodontal sites. The median total bacterial concentration of the periodontal sites was four times higher than that detected in the control sites (*P* < 0.001). Five pathobionts showed significantly higher concentrations in the periodontal sites than the control sites. These species were: *Fn, Pm, Pg*, *Tf*, and *Td* (*P* < 0.001 for all the bacteria except *Fn*, *P* = 0.023). In terms of the 127 bacterial clusters, all except *PiAaFn*, *PiFn*, and *PiAa* showed significantly higher concentrations in the periodontal sites than the control sites (*P*-values ranged from <0.001 to 0.009). After applying the Benjamini–Hochberg correction, all the significant *P*-values were maintained (**Table 3** and **Supplementary Data Sheet S1**). With regard to smoking habit, no significant differences were found in the detection frequencies or the bacterial concentrations for each subgingival site between the smokers and non-smokers.

**Table 3:** Comparison of the detection frequencies and concentrations (pg/μl) of seven periopathogens between the two subgingival sites (control and periodontal sites) in the periodontal patient group.

Bacteria	Detection frequency^1^; No. of sites (Percentage)			Concentration (pg/μl); Mean ± SD, Median (IQR)		
	Control site	Periodontal site	*P*-value	Control site	Periodontal site	*P*-value^3^
Total bacteria^2^	40 (100)	40 (100)	NA	318.46 ± 427.68	1006.05 ± 915.18	<0.001
				184.05 (429.89)	773.88 (1066.76)
*Aa*	7 (17.5)	7 (17.5)	NS	1.17 ± 5.35	2.27 ± 9.95	NS
				0.00 (0.00)	0.00 (0.00)
*Fn*	37 (92.5)	39 (97.5)	NS	9.32 ± 29.82	22.04 ± 49.73	0.023
				0.02 (0.05)	0.03 (5.71)
*Pm*	40 (100)	40 (100)	NA	22.08 ± 23.16	47.69 ± 41.32	<0.001
				13.16 (31.55)	35.83 (45.59)
*Pg*	36 (90)	36 (90)	NS	113.25 ± 69.34	373.13 ± 1.23	<0.001
				7.74 (79.67)	209.71 (498.89)
*Pi*	29 (72.5)	26 (65)	NS	25.62 ± 57.27	37.23 ± 81.54	NS
				0.01 (5.62)	0 (38.22)
*Tf*	40 (100)	40 (100)	NA	139.04 ± 204.93	486.04 ± 5.75	<0.001
				82.21 (198.00)	251.79 (562.65)
*Td*	33 (82.5)	32 (80)	NS	7.99 ± 24.28	37.66 ± 58.82	<0.001
				0.69 (4.06)	9.63 (49.54)

*IQR, interquartile range; NA, not applicable; NS, not significant; *Aa, A. actinomycetemcomitans; Fn, F. nucleatum; Pm, P. micra; Pg, P. gingivalis; Pi, P. intermedia; Tf, T. forsythia; Td, T. denticola.*^1^The threshold value for detection frequency was 10–100 copies of DNA (3–5 femtograms/10^-*15*^ grams). ^2^The variable ‘total bacteria’ represents the sum of the concentrations of the seven bacteria analyzed. ^3^After applying the Benjamini–Hochberg correction, all the *P*-values that showed significance were maintained*.

### Multivariate Predictive Modeling of Chronic Periodontitis at Site-Specific Level Based on Subgingival Pathobiont Levels: Selection and Validation of Models, and the Development of Nomograms

A first description of the relation between pathobiont levels, as well as with the clinical parameters (PPD, CAL) is given in **Supplementary Data Sheet S1**, by means of their Spearman correlations. Almost all correlations between bacteria and with the PPD and CAL values were positive. The interpretation is that when a subgingival site with periodontal destruction was present, the majority of pathobionts presented larger values, which can be verified in the comparative analysis between control and periodontal sites shown in **Table 3**. However, the correlation values between the bacteria and these with the clinical parameters were not very high, the strongest associations being observed between *Pg* and *Tf* (Rho = 0.699) and several clusters with ≥4 bacteria and CAL (Rho > 0.540) (**Supplementary Data Sheet S1**).

Applying the statistical criteria set out in the section ‘Materials and Methods,’ a total of eight models were selected (**Table 4** and **Supplementary Data Sheet S2**). In all of them, the predictor variables were represented by one bacterial cluster composed of at least three species. The most common bacteria were: *Tf* and *Pi* (both were present in eight models), followed by *Td* and *Fn* (both were present in six models) and *Aa* and *Pm* (both were present in five and three models, respectively). The concentration values of the optimal threshold ranged from 218.86 pg/μl for the *PiTfFn* model to 264.16 pg/μl for the *TdPiTfAaFnPm* model. There was no predictive model formed by two bacterial clusters that fulfilled the established statistical criteria.

**Table 4:** Description of the eight models based on bacterial clusters, including the apparent and bias-corrected AUC values, as well as optimal threshold values.

Cluster-based model	AUC	bc-AUC	Optimal threshold^1^
-0.98148 + 0.00329*PiTfFn*	0.773	0.772	218.86
-1.0115 + 0.00331*TdPiTfAa*	0.785	0.784	220.61
-1.03208 + 0.00325*TdPiTfFn*	0.783	0.781	218.27
-0.98534 + 0.00328*PiTfAaFn*	0.773	0.774	220.71
-1.03627 + 0.00324*TdPiTfAaFn*	0.783	0.781	220.14
-1.10445 + 0.00326*TdPiTfAaPm*	0.787	0.784	264.45
-1.12387 + 0.00321*TdPiTfFnPm*	0.788	0.786	262.26
-1.12778 + 0.00320*TdPiTfAaFnPm*	0.789	0.790	264.16

^1^*The best cut-off value of bacterial concentration for each model (optimal threshold) was determined so that the percentage of correct predictions was the maximum*.

The main results on the apparent and bc-measures of discrimination and classification of eight pathobiont-based models are detailed in **Table 5** and **Supplementary Data Sheet S2**. The apparent AUC values of these models ranged from 0.773 to 0.789. The *PiTfFn* cluster showed an apparent AUC value of 0.773 (sensitivity and specificity = 75.0%; DOR = 9.0). When *Td* was incorporated in this cluster, a new predictive model emerged with better apparent AUC, sensitivity and DOR values (0.783, 77.5%, and 10.3, respectively). When *Pm* and *AaPm* were incorporated in the *TdPiTfFn* cluster, we obtained the two best predictive models with apparent AUC values of 0.788 and 0.789, respectively, as well as sensitivity and specificity values of 77.5% and DOR of 11.8 for both models. However, when *Aa* and *TdAa* were incorporated in the *PiTfFn* cluster, the two predictive models that appeared did not have better discrimination and classification measures than those detected in clusters of lower numbers of bacteria, such as *PiTfFn* and *TdPiTfFn.*

**Table 5:** Measures of discrimination and classification of the eight models based on bacterial clusters.

Model	ACC (%)	Sensitivity (%)	Specificity (%)	Positive predictive value (%)	Negative predictive value (%)	Diagnostic OR
*PiTfFn*	75.0 72.4	75.0 72.6	75.0 72.8	75.0 72.4	75.0 73.0	9.0 7.1
*TdPiTfAa*	75.0 71.9	75.0 72.0	75.0 72.6	75.0 72.1	75.5 73.0	9.0 6.8
*TdPiTfFn*	76.2 73.4	77.5 74.8	75.0 72.8	75.6 73.0	76.9 74.5	10.3 7.9
*PiTfAaFn*	75.0 72.6	75.0 72.8	75.0 73.1	75.0 72.3	75.5 73.8	9.0 7.2
*TdPiTfAaFn*	76.2 73.4	77.5 74.5	75.0 73.1	75.6 73.1	77.2 74.8	10.3 7.9
*TdPiTfAaPm*	77.5 75.0	75.0 72.5	80.0 78.1	79.0 76.6	76.1 74.1	12.0 9.4
*TdPiTfFnPm*	77.5 74.9	77.5 74.9	77.5 75.6	77.7 75.3	77.7 75.5	11.8 9.2
*TdPiTfAaFnPm*	77.5 75.3	77.5 75.2	77.5 75.9	77.7 75.6	77.5 75.8	11.8 9.6

*In each cell, the first value is referred to the apparent performance measures and the second, to the corrected performance measures (bc-ACC, bc-sensitivity, bc-specificity, bc-PPV, bc-NPV, and bc-DOR) by the level of optimism calculated using a bootstrap procedure. The 95% CIs of the different performances measures, excepting DOR, are detailed in **Supplementary Data Sheet S2***.

The *TdPiTfAa* cluster showed an apparent AUC value of 0.785 (sensitivity and specificity = 75.0%; DOR = 9.0). When *Pm* was incorporated in this cluster, a new predictive model appeared with better apparent AUC, specificity and DOR values (0.787, 80.0%, and 12.0, respectively). The optimism values obtained by the bootstrap methods in relation to the performance measures of these models ranged from -0.001 to 0.003 in the AUC values and 1.52–3.07% in the classification indices.

**FIGURE 3:**
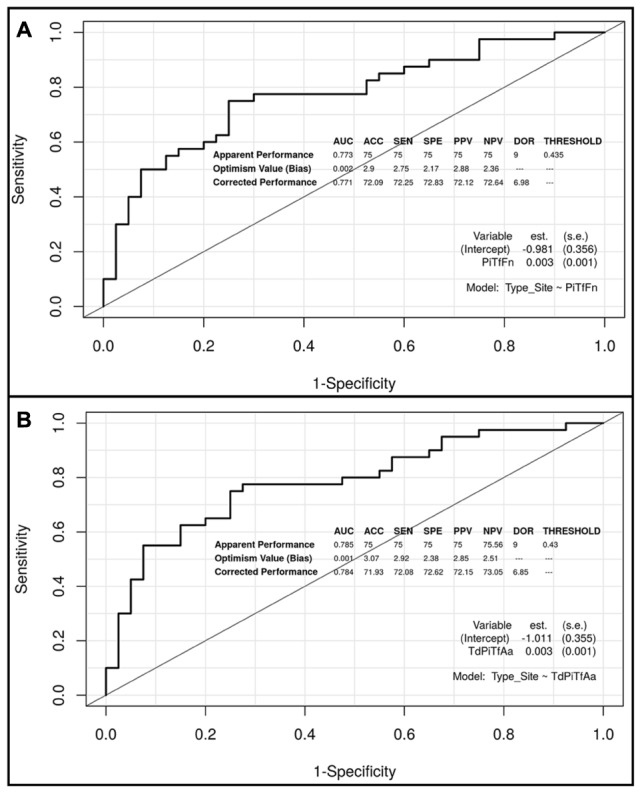
Receiver operating characteristic (ROC) curves of the two predictive models based on the *PiTfFn* and *TdPiTfAa* clusters, including the apparent and bias-corrected measures by bootstrapping. **(A)** *PiTfFn* cluster; **(B)** *TdPiTfAa* cluster.

**FIGURE 4:**
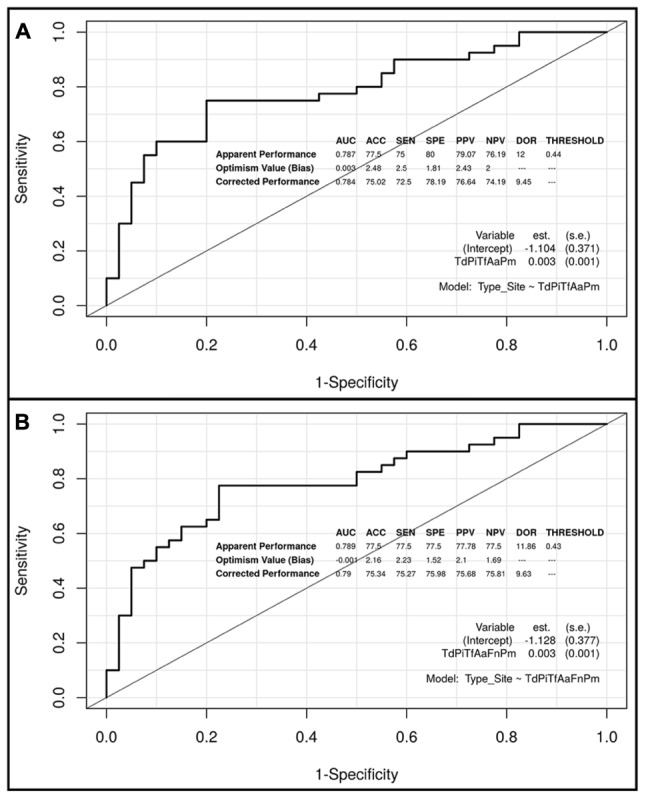
Receiver operating characteristic (ROC) curves of the two predictive models based on the *TdPiTfAaPm* and *TdPiTfAaFnPm* clusters, including the apparent and bias-corrected measures by bootstrapping. **(A)** *TdPiTfAaPm* cluster; **(B)** *TdPiTfAaFnPm* cluster.

**FIGURE 5:**
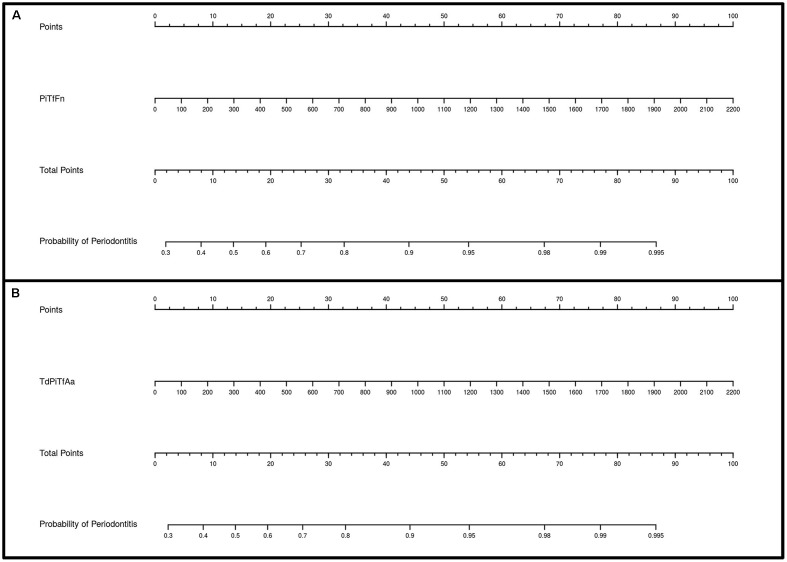
Diagnostic nomograms based on the *PiTfFn* and *TdPiTfAa* models for predicting the probability of having a periodontal site. The probability of having a subgingival site with periodontal destruction is calculated by drawing a line to the point on the axis for each of the following variables: **(A)** *PiTfFn* cluster; **(B)** *TdPiTfAa* cluster. The points for each variable are located on the total point line. Next, a vertical line is projected from the total point line to the predicted probability bottom scale to obtain the individual probability of periodontitis at the site-specific level.

**FIGURE 6:**
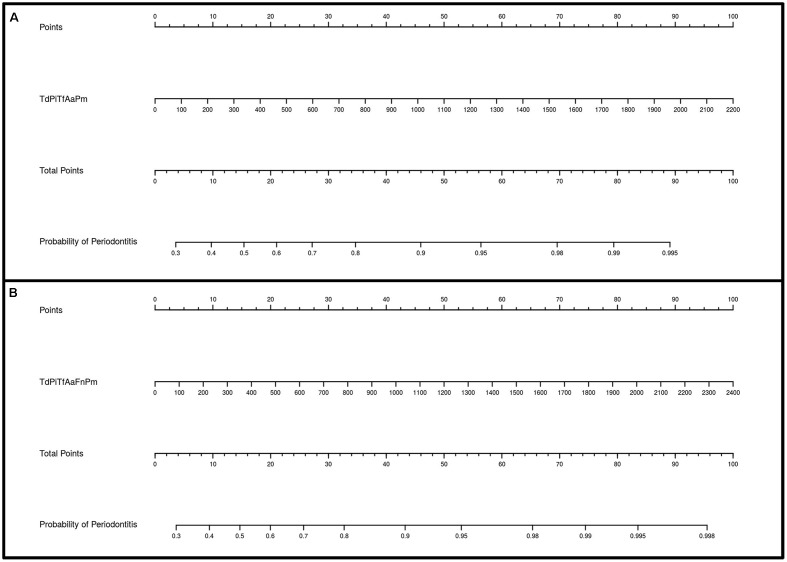
Diagnostic nomograms based on the *TdPiTfAaPm* and *TdPiTfAaFnPm* models for predicting the probability of having a periodontal site. The probability of having a subgingival site with periodontal destruction is calculated by drawing a line to the point on the axis for each of the following variables: **(A)** *TdPiTfAaPm* cluster; **(B)** *TdPiTfAaFnPm* cluster. The points for each variable are located on the total point line. Next, a vertical line is projected from the total point line to the predicted probability bottom scale to obtain the individual probability of periodontitis at the site-specific level.

**Figures 3**, **4** show the ROC curves of the most predictive clusters with the lowest number of species – *PiTfFn* and *TdPiTfAa* – and the highest number of bacteria – *TdPiTfAaFnPm* and *TdPiTfAaPm*. **Figures 5**, **6** show the diagnostic nomograms of the four previous models. As noted previously, the discrimination and classification performance values of all the nomograms were high, indicating their good accuracy. Overall, in eight nomograms, the high concentration levels of the different bacterial clusters were associated with an increased probability of having a subgingival site with periodontal destruction in a patient with chronic periodontitis. Supplementary Files show these graphics of the eight models with an AUC ≥0.76 and sensitivity and specificity values ≥ 75% (**Supplementary Data Sheets S3**, **S4**, and Figures S1–S8).

## Discussion

### Methodological Aspects of the qPCR Studies

In terms of study design, a control group (healthy subjects) is compared to a group of periodontitis patients in numerous qPCR studies of periodontal pathogens at the subgingival level (Braga et al., 2010; Kretschmar et al., 2012). The control group may also include gingivitis patients (Scapoli et al., 2015), or they may constitute a separate group (Masunaga et al., 2010). In addition, there are other authors who have studied subgingival samples microbiologically using the qPCR from different periodontal patient groups according to the degree of periodontal affection (mild, moderate, or severe) (Zorina et al., 2011) or the type of disease (chronic vs. aggressive) (Casarin et al., 2010). There are also qPCR studies that have been performed exclusively on subjects with periodontitis (Kotsilkov et al., 2015; Milne et al., 2015).

Although certain subject-associated factors can increase an individual’s overall susceptibility to periodontal diseases, it appears that not all sites within a subject are equally susceptible at one time (Griffiths, 2003). This site-specific clinical pattern can be attributed to differences in the bacterial species composition of subgingival plaque (Byrne et al., 2009). However, there is little evidence in the literature of the influence of pathobionts analyzed by the qPCR in relation to the site-specific severity of chronic periodontitis (Mineoka et al., 2008; Teixeira et al., 2009; Al-hebshi et al., 2014). As a consequence, we propose using a qPCR technique to study the relationship between pathobiont levels from subgingival sites with different degrees of disease (control sites vs. periodontal sites) within the same periodontal patient. This methodology (paired design) means that the effects of inter-individual variations in factors other than bacterial composition are avoided.

Regarding the species studied, we decided to quantify them initially according to Socransky’s bacterial complexes (Socransky et al., 1998), with those most frequently studied being: three species of the Red Complex (*Pg, Tf*, and *Td*), the main three species of the Orange Complex (*Fn*, *Pi*, and *Pm*) and a species from the Green Complex (*Aa*). Consequently, our intention was to focus on various well-known pathobionts that could be used as biomarkers in a possible diagnostic tool for dental practitioners in order to improve disease control and reduce the future risk of progression seen in everyday clinical practice.

In numerous qPCR papers, the authors report the number of DNA copies as a measure of the quantification of the bacterial load rather than cell numbers, because the copy number of the marker gene, particularly the 16S rRNA gene, varies from one species to another and is unknown for some of them (Al-hebshi et al., 2014, 2015; Yang et al., 2016). This implies that available bacterial counts for some of the tested genera/species have probably been over-estimated (Al-hebshi et al., 2014). In the present series, we used a new qPCR technique with TaqMan probes and Cod-UNG enzymes for the molecular detection of these pathobionts. This patented technique allows the easy design of probes for the detection of any pathogen while maintaining excellent sensitivity and specificity (Bou et al., 2014). In the present study, the absolute quantification of the DNA of each species was performed using the standard curve method. Moreover, the target genes studied were different single-copy genes per genome, which may allow absolute counts of DNA copies to be reported in terms of cell numbers.

From an analytical point of view, the majority of authors in their respective qPCR studies applied a ‘simple approach.’ As a result, the subgingival levels of pathobionts in control volunteers and periodontal patients were compared for testing using q-PCR, whether or not a particular bacterium is absent/present and/or diminished/elevated at the individual level (Braga et al., 2010; Polonyi et al., 2013; Kotsilkov et al., 2015; Milne et al., 2015; Scapoli et al., 2015; Golynska et al., 2016). In parallel with current trends in big data analytics for genomic medicine (He et al., 2017), further evidence is required about applying multivariate analytical approaches to qPCR data. This would enable there to be a better understanding of the complex bacterial interactions present in chronic periodontitis. This type of analytical approach was used in our study.

### Detection Frequencies and Levels of Pathobionts in the Severity of Chronic Periodontitis: Control Sites vs. Periodontal Sites

Al-hebshi et al. (2014) used the qPCR to investigate seven pathobionts (the species of the Red Complex, the genera *Fusobacterium* spp. and *Prevotella* spp., as well as *Aa*) in healthy and periodontal subgingival sites within the same periodontal patient. This study was similar to ours methodologically. The Al-hebshi’s series revealed detection percentages of 100% for *Pm*, *Td*, *Tf*, *Fusobacterium* spp. and *Prevotella* spp., 97.5% for *Pg*, and 67.5% for *Aa*. We agree with them on the high detection rates found for the Red Complex species, *Fn* and *Pm* (80–100%), confirming that the mere presence of the bacteria is not an indicator of the clinical situation (Byrne et al., 2009; Charalampakis et al., 2013). However, in contrast to Al-hebshi et al. (2014), our series of periodontal patients showed lower detection frequencies for *Pi* (69%) and, especially, *Aa* (17.5%), although those values were not conditioned by the subgingival site tested. Initially, our results on the low prevalence of *Aa* confirmed its marginal role in chronic periodontitis in our study population, which reflects the findings of other authors (Gatto et al., 2014); however, we will later see how its role changes in the multivariate analysis.

In Al-hebshi’s study (Al-hebshi et al., 2014), the levels of *Pm*, *Prevotella* spp*., Pg*, *Tf*, and *Td* were higher in subgingival sites with periodontal destruction than the control sites. However, after correcting for multiple comparisons, these differences only remained statistically significant for *Pm* and *Tf.* This was probably due to the small sample size (only 20 periodontal patients). In the present study, our results revealed that periodontal sites had significantly higher concentrations for the three Red Complex species (concentration increments of 27, 14, and 3 times for *Pg*, *Td*, and *Tf*, respectively) and *Pm* and *Fn* (concentration increments of 2.7 and 1.5 times, respectively).

Our findings demonstrated that all the pathobionts (except *Aa* and *Pi*) were present at the control sites (non-active sites) and that an increase in their concentrations was required to trigger activity in the periodontal site; *Pg* was the bacteria that had a higher increment. The relevance of the Red Complex species together and *Pg* alone in the pathogenesis of periodontitis has been proved in numerous *in vitro* studies (Bao et al., 2014; Lin et al., 2014; Willi et al., 2014). A bacterial consortium named the Orange Complex, which includes *Fn* and *Pm*, was indicated as preceding the Red Complex with respect to colonization and proliferation (Socransky et al., 1998). In relation to *Fn* and *Pm*, there is growing evidence based on *in vitro* research on their role as periodontal pathogens (Nonnenmacher et al., 2003; Lee and Baek, 2013; Wang et al., 2013; Kim and Lee, 2014; Marchesan et al., 2016).

Although evaluating the influence of smoking was not a major objective of the present study, our findings in this regard are consistent with those of Tomita et al. (2013), as we did not detect significant differences in the profiles of the seven species present in the sampled subgingival sites (control and periodontal sites). These sites showed similar clinical characteristics between smokers and non-smokers. Nevertheless, these results should be interpreted with caution since, in larger series, other authors have found significantly higher prevalences of *Td* and *Tf* and significantly higher levels of *Aa*, *Pg*, and *Tf* in smokers with chronic periodontitis (Gatto et al., 2014; Guglielmetti et al., 2014).

### Multivariate Predictive Modeling of the Subgingival Levels of Pathobionts: Selection of the Best Models and the Development of Nomograms

In periodontology, traditional clinical criteria are often inadequate for determining sites of active disease, measuring the degree of susceptibility to future progression, and monitoring the response to therapy (Giannobile et al., 2009; Korte and Kinney, 2016). In this sense, there is a need for research on innovative diagnostic tests based on biomarkers that focus on the early recognition of the microbial challenge to the host, providing a benefit in terms of disease control at the site-specific level.

The first step to assess whether a test could be clinically useful is the analysis of its predictive ability (Deeks, 1999). In this sense, there are some papers in the literature that have focused on the analysis of the predictive ability of the pathobiont levels quantified by the qPCR to distinguish different degrees of periodontal affection in subgingival samples from different individuals (Ramseier et al., 2009; Saygun et al., 2011; Kinney et al., 2014; Al-hebshi et al., 2015; Torrungruang et al., 2015). Several of these studies coincide in terms of detecting that the subgingival levels of certain bacteria, such as the Red Complex species, *C. rectus*, *Pi*, *Pm* and even a new bacterial phylum of oral Synergistetes, showed a good diagnostic power for distinguishing between healthy/gingivitis patients and those with periodontitis (AUC values ranged from >0. 74 to >0.80) (Ramseier et al., 2009; Al-hebshi et al., 2015).

However, we have found very few papers like ours on bacteria-based predictive models for diagnosing periodontitis severity or progression using a paired design (Byrne et al., 2009; Nomura et al., 2012; Charalampakis et al., 2013; Gatto et al., 2014). Charalampakis et al. (2013) identified sites at risk of future progression during 2 years of maintenance in 50 patients with chronic periodontitis. This research was based on longitudinal clinical and microbiological monitoring using DNA–DNA hybridization involving 25 bacterial species. In this series, both members of the Red Complex as well as of the ‘B Complex’ (*Pi*, *Fn* and *C. rectus*) were equally able to identify periodontitis from non-periodontitis sites and severe periodontitis sites from non-severe periodontitis sites; these bacterial complexes had AUC values above 0.75. In accordance with these results (Charalampakis et al., 2013), in our study the best predictive models with AUC values ≥0.76 (sensitivity and specificity ≥75%), which could be regarded as good (Hosmer et al., 2013), included the concentration levels of different bacterial clusters formed by at least three bacteria. These findings were ratified by the analysis of the internal validation that was performed, since the optimism values obtained by the bootstrap techniques were not high. Therefore, in accordance with the findings of previous authors (Byrne et al., 2009; Charalampakis et al., 2013), our results confirm that the use of bacterial quantitative data rather than dichotomous data (presence/absence) provides more statistical power for detecting any predictive relationship with disease severity.

Interestingly, and unlike Charalampakis’s study, the bacteria present in all the predictive clusters were *Tf* and *Pi* (both in eight models), followed by *Td* and *Fn* (both in six models) and *Aa* and *Pm* (both in five and three models, respectively). All these clusters were constituted by bacteria that belong to different Socransky’s complexes and have different pathogenic roles in disease severity in terms of individual concentration levels. Such an outcome was previously observed in our own series. In fact, although *Pi* and *Aa* had non-significant, higher concentrations in the periodontal sites than the control sites, their levels contributed to increasing the predictive ability of the cluster in which they were incorporated. On the other hand, although *Pg* was the species that showed the highest concentration increment in the periodontal sites, surprisingly this species did not have a high power for predicting periodontitis severity in comparison with other pathobionts, since it was not included in any of the best predictive models. From a predictive perspective, these findings contribute to *Pg* being regarded as a ‘keystone’ pathobiont which is a microorganism that can change the environment to alter proportions or levels of other symbionts and pathobionts within the ecological niche; these microorganisms are the main ones responsible for triggering the destructive cascade that provokes the activation of inflammation and subsequent bone destruction (Costalonga and Herzberg, 2014).

In accordance with previous authors (Byrne et al., 2009; Charalampakis et al., 2013), we demonstrated that the detection of a specific threshold of concentration levels of certain bacterial clusters may serve as a predictor of periodontitis severity at site-specific level. Two main predictive clusters were identified: *PiTfFn* (AUC = 0.773; sensitivity and specificity = 75.0%; DOR = 9.0) and *TdPiTfAa* (AUC = 0.785; sensitivity and specificity = 75.0%; DOR = 9.0). In relation to *PiTfFn*, when other bacteria or clusters such as *Td*, *Pm*, and *AaPm* were incorporated in this cluster, new predictive models emerged with better predictive parameters. The two best predictive clusters were *TdPiTfFnPm* and *TdPiTfAaFnPm*, with AUC values of 0.788 and 0.789, respectively (sensitivity and specificity values of 77.5% and DOR of 11.8 for both models). In relation to *TdPiTfAa*, when *Pm* was incorporated in this cluster, a new predictive model appeared with better AUC, specificity and DOR values (0.787, 80.0%, and 12.0, respectively).

To the best of our knowledge, this study presents the first evidence that these new bacterial clusters are capable of predicting the severity of chronic periodontitis, with several species detected outside the Red Complex that contribute to the detection of periodontal destruction at the specific-site level. To date, the findings obtained by open-ended molecular approaches support the hypothesis that chronic periodontitis is initiated by polymicrobial synergy and the dysbiosis of the entire microbial community (PSD model). The condition is characterized not only by a greater involvement of the ‘established’ pathobionts, as studied in this series, but also by the coexistence of other pathobionts with an unknown role such as *Anaeroglobus*, *Bulleidia*, *Desulfobulbus*, *Filifactor alocis*, *Mogibacterium*, *Phocaeicola*, *Schwartzia, or TM7* (Camelo-Castillo et al., 2015a,b). Given this new pathogenic approach of the disease, it would be very interesting to study the predictive ability of these new pathobionts and other symbionts in chronic periodontitis, as well as their relationship with host mediators.

Nomograms are simplified representations of complicated statistical models, and their clinical value relates to the fact that they map the predicted probabilities into points on a scale from 0 to 100 in a user-friendly graphical interface (Iasonos et al., 2008). To our knowledge, this is the first study providing several nomograms based on the concentration levels of certain bacterial clusters to predict the probability of having a periodontal site in a patient with chronic periodontitis.

Our nomograms, which are derived from the eight best-fitting models, fulfilled the requirements of discrimination. Overall, in all the nomograms, higher concentration levels of different bacterial clusters were associated with an increased probability of having a periodontal site in a periodontal patient. The use of only a few variables is desirable in nomograms to increase their utility in clinical practice (Altman and Royston, 2000). Consequently, of the nomograms developed in the present study, we highlight those based on the clusters formed by the lower number of species, *PiTfFn* and *TdPiTfAa*. The application of these tools in the field of clinical activity would improve the identification of sites with periodontal destruction that are at possible risk of future progression, contributing to the decision-making process and treatment planning dilemmas.

Our research has some limitations. The most important weakness is that the prediction of the study’s accuracy is only measured in the samples that generated the model equations. As a consequence, to evaluate the reproducibility of the models and control for the possibility of overfitting, we validated the prediction rule internally (discrimination and classification measures) using bootstrap methods on the original derivation dataset by sampling with replacements for 1000 iterations (Steyerberg et al., 2001a,b). This internal validation method revealed optimal results on the control of overfitting in the models.

The evaluation of these cluster-based predictive models and nomograms is a potential future research direction. Firstly, it would greatly benefit the strength of our study if the predictive accuracy of the predictive models derived from our series could be measured in a large ‘external’ or independent cohort of patients to verify whether our findings are universally applicable or if they are conditioned by differences associated with geographic regions and ethnic groups (Herrera et al., 2008; Wara-aswapati et al., 2009; Psoter et al., 2011). An appropriate calibration analysis of the predictive models should also be performed. Secondly, the potential prognostic value at the site-specific level of these cluster-based predictive models with regard to disease progression and the response to treatment in periodontal patients should be exploited in longitudinal studies, as should their relationship with host mediators and their potential predictive accuracy in saliva samples.

## Conclusion

We corroborated the important etiopathogenic role in quantitative terms, not only of Socransky’s Red Complex pathobionts, but also of other species such as *Parvimonas micra*, in the severity of chronic periodontitis at site-specific level. Several statistically validated models based on the bacterial concentration levels with a good predictive accuracy demonstrated that some species are good biomarkers when it comes to distinguishing a site with periodontal destruction in a periodontal patient. All these models consist of different clusters formed by several bacteria, at least three, which belong to different Socransky’s complexes. The most predictive clusters with the lowest number of species were *PiTfFn* and *TdPiTfAa*, and those with the highest number were *TdPiTfFnPm* and *TdPiTfAaFnPm.* In all the nomograms, higher concentration levels of these clusters were associated with an increased probability of finding a periodontal site in a patient with chronic periodontitis. The clinical implications of these predictive tools could include improved patient monitoring and the control of disease activity at the site-specific level. However, additional evidence is needed to test the external validity of these bacterial cluster-based models for predicting chronic periodontitis severity at the site-specific level and confirming the clinical value of the proposed nomograms.

## Author Contributions

IT, AR-I, NA-B, and LN participated in the recruitment and diagnosis of patients, the collection of oral samples, in reviewing the literature and writing the paper. ML and MT participated in the quantification by qPCR of pathobionts and in the interpretation of the obtained results. IT and CB-C participated in the statistical design of the models, in the internal validation analysis and the development of the graphics. All authors made substantial contributions to all of the following: (1) the conception and design of the study, or acquisition of data, or analysis and interpretation of data, (2) drafting the article or revising it critically for important intellectual content, (3) the review and approval of the manuscript in its submitted form.

## Conflict of Interest Statement

The authors declare that the research was conducted in the absence of any commercial or financial relationships that could be construed as a potential conflict of interest.

## Supplementary Material

DATA SHEET S1 Dataframe and univariate analysis.

DATA SHEET S2 Description of cluster-based models with an AUC value ≥0.76 and sensitivity and specificity values ≥75%, and their principal characteristics. Results of the analysis on the internal validation by bootstrap methods of these predictive models.

DATA SHEET S3 (Figures S1–S8): Receiver operating characteristic (ROC) curves of the cluster-based models with an AUC ≥0.76 and sensitivity and specificity values ≥75%.

DATA SHEET S4 (Figures S1–S8): Diagnostic nomograms derived from the cluster-based models with an AUC ≥0.76 and sensitivity and specificity values ≥75%.
