# Supplementary material for: Quantification by qPCR of Pathobionts in Chronic Periodontitis: Development of Predictive Models of Disease Severity at Site-Specific Level
Source: Front Microbiol. 2017 Aug 9;8:1443. doi: 10.3389/fmicb.2017.01443 (PMC5552702; doi:10.3389/fmicb.2017.01443)
Supplement: DATA SHEET S3 — (Figures S1–S8): Receiver operating characteristic (ROC) curves of the cluster-based models with an AUC ≥0.76 and sensitivity and specificity values ≥75%. [file Data_Sheet_3.pdf]

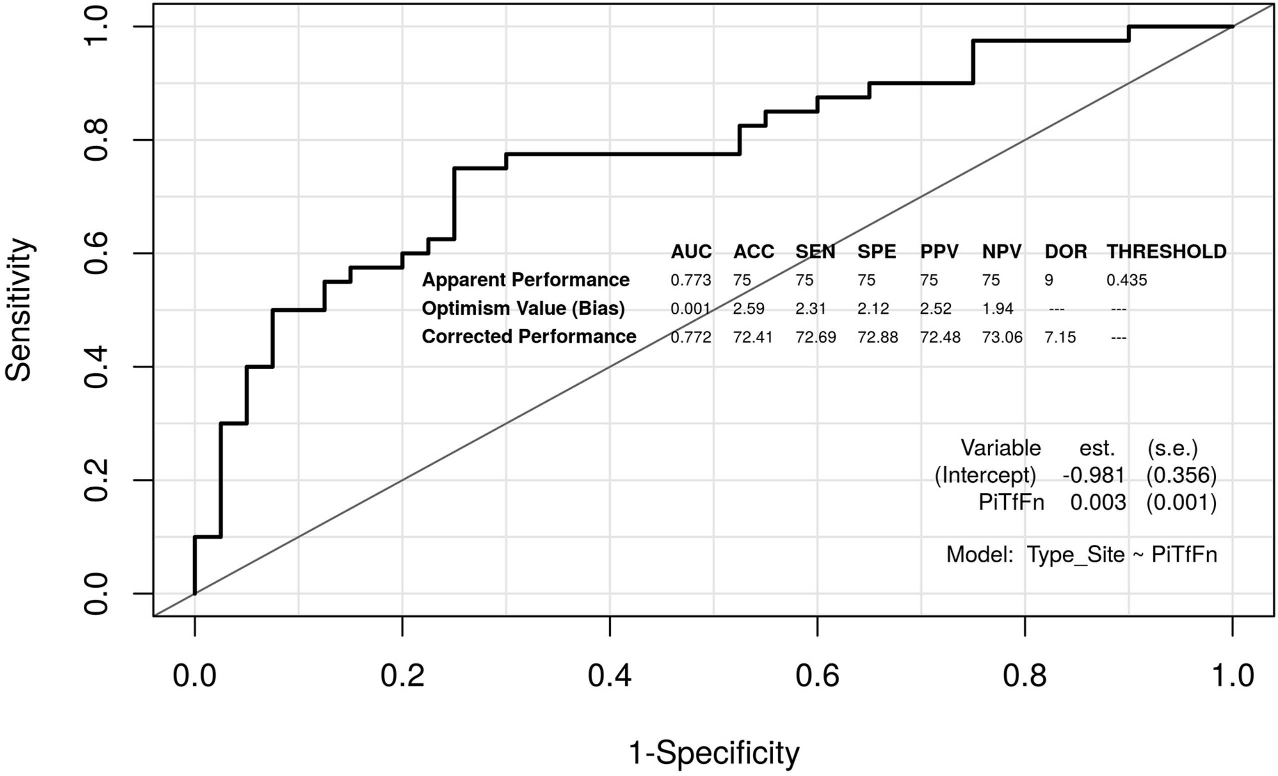

Sensitivity
1-Specificity
0.0
0.2
0.4
0.6
0.8
1.0
AUC ACC SEN SPE PPV NPV DOR THRESHOLD
Apparent Performance 0.773 75 75 75 75 75 9 0.435
Optimism Value (Bias) 0.001 2.59 2.31 2.12 2.52 1.94 --- ---
Corrected Performance 0.772 72.41 72.69 72.88 72.48 73.06 7.15 ---
Variable est. (s.e.)
(Intercept) -0.981 (0.356)
PiTfFn 0.003 (0.001)
Model: Type_Site ~ PiTfFn

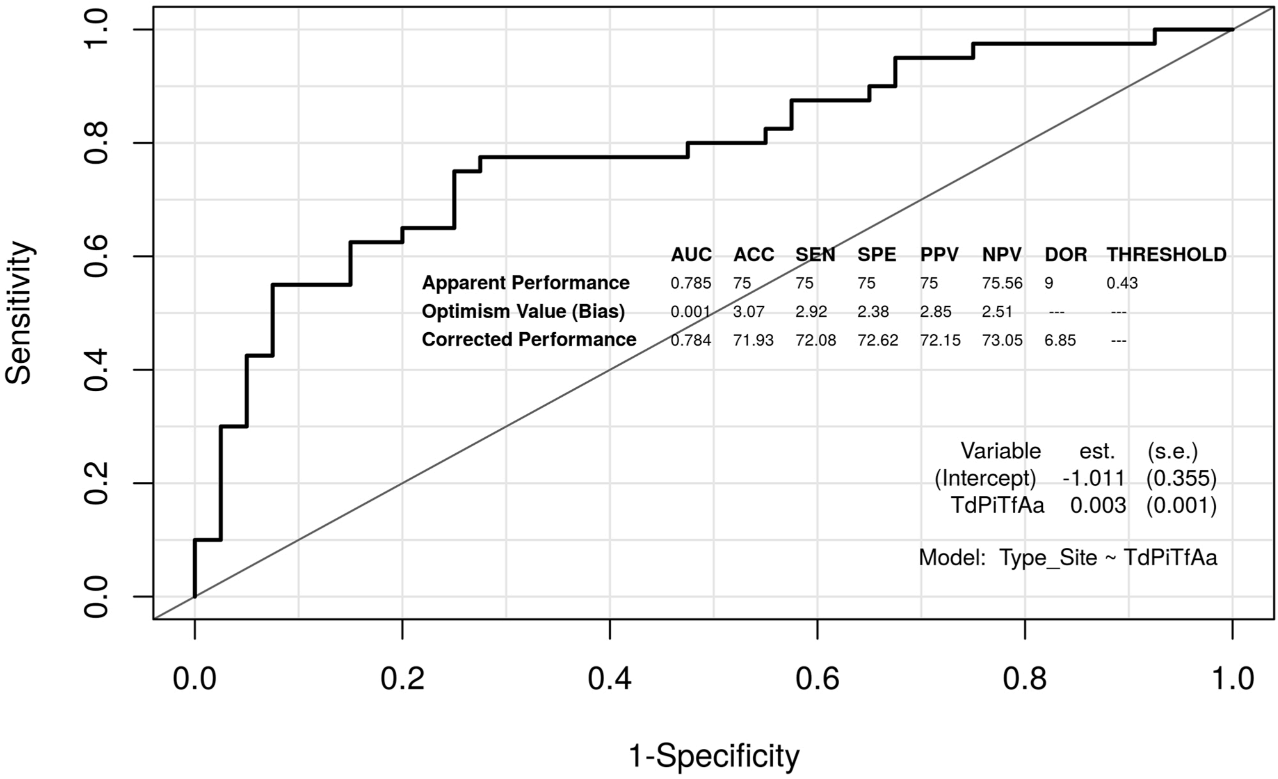

Sensitivity
1-Specificity
0.0
0.2
0.4
0.6
0.8
1.0
AUC ACC SEN SPE PPV NPV DOR THRESHOLD
Apparent Performance 0.785 75 75 75 75 75.56 9 0.43
Optimism Value (Bias) 0.001 3.07 2.92 2.38 2.85 2.51 --- ---
Corrected Performance 0.784 71.93 72.08 72.62 72.15 73.05 6.85 ---
Variable est. (s.e.)
(Intercept) -1.011 (0.355)
TdPiTfAa 0.003 (0.001)
Model: Type_Site ~ TdPiTfAa

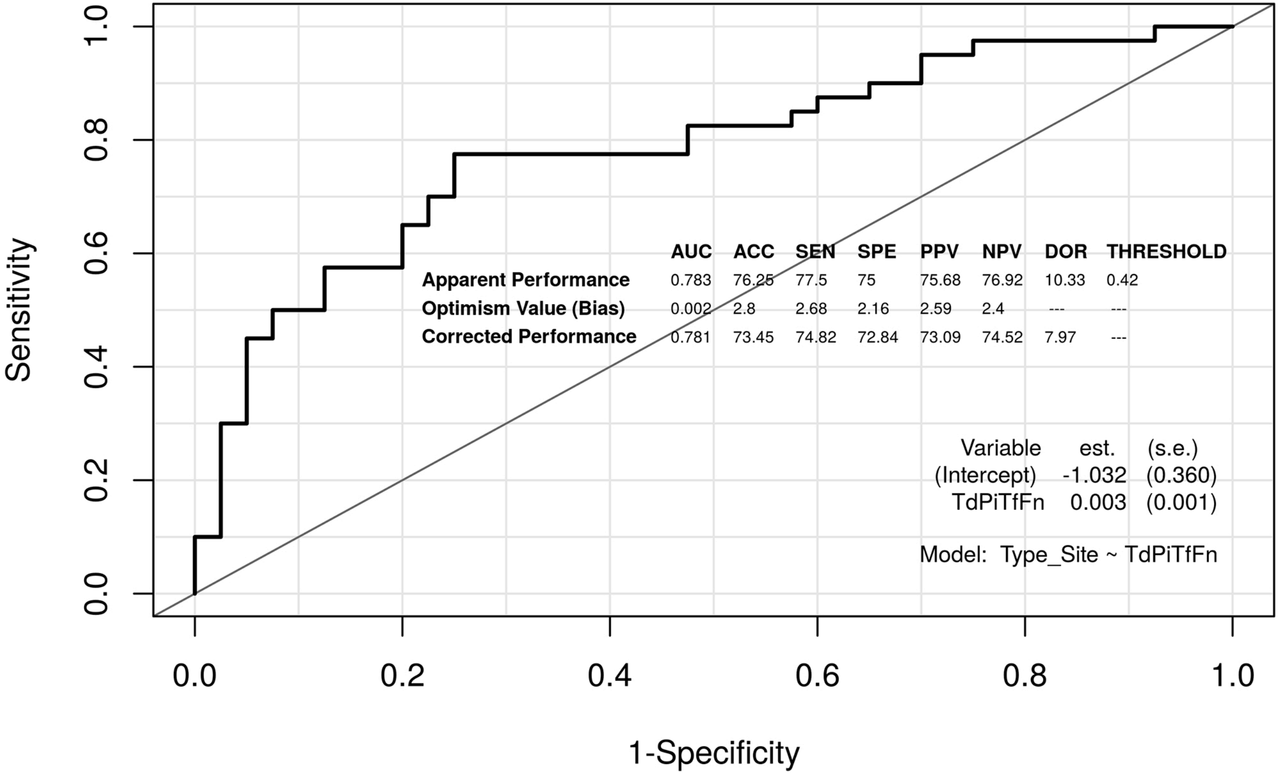
Sensitivity
1-Specificity
0.0
0.2
0.4
0.6
0.8
1.0
AUC ACC SEN SPE PPV NPV DOR THRESHOLD
Apparent Performance 0.783 76.25 77.5 75 75.68 76.92 10.33 0.42
Optimism Value (Bias) 0.002 2.8 2.68 2.16 2.59 2.4 --- ---
Corrected Performance 0.781 73.45 74.82 72.84 73.09 74.52 7.97 ---
Variable est. (s.e.)
(Intercept) -1.032 (0.360)
TdPiTfFn 0.003 (0.001)
Model: Type_Site ~ TdPiTfFn

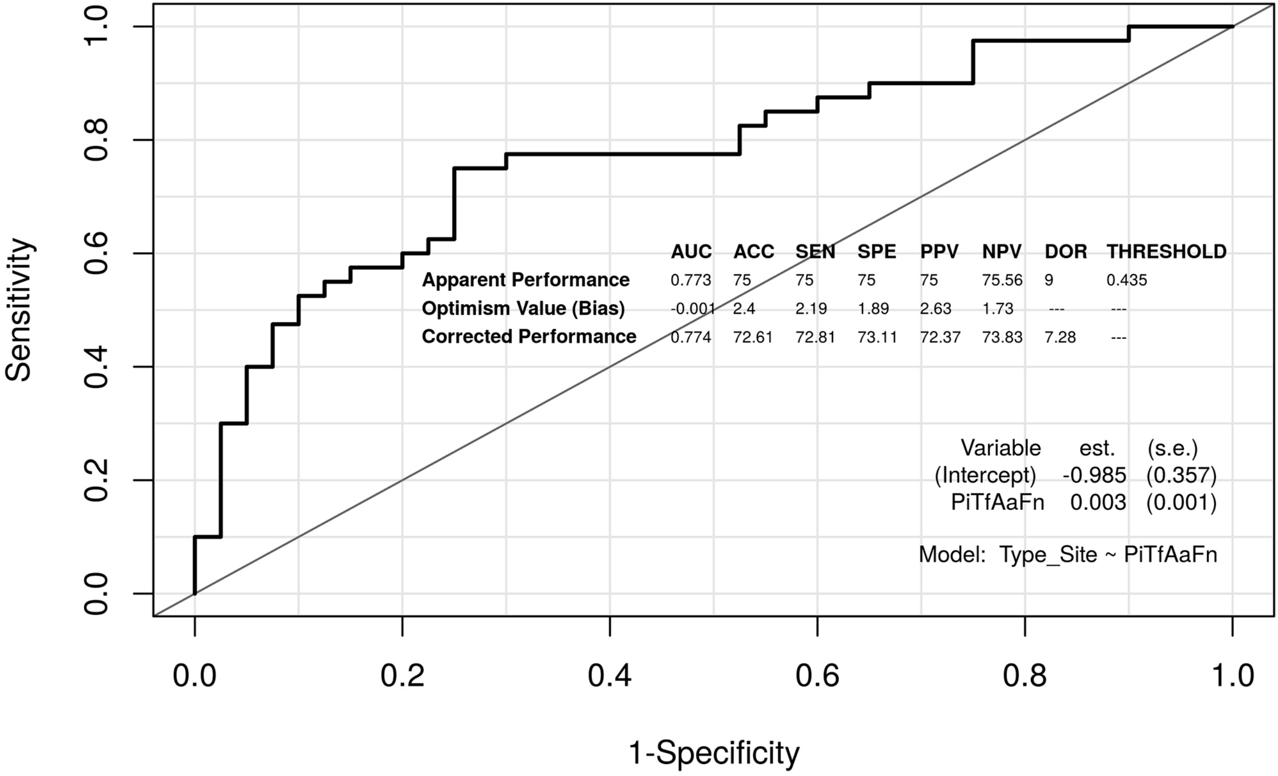

Sensitivity
1-Specificity
0.0
0.2
0.4
0.6
0.8
1.0
AUC ACC SEN SPE PPV NPV DOR THRESHOLD
Apparent Performance 0.773 75 75 75 75 75.56 9 0.435
Optimism Value (Bias) -0.001 2.4 2.19 1.89 2.63 1.73 --- ---
Corrected Performance 0.774 72.61 72.81 73.11 72.37 73.83 7.28 ---
Variable est. (s.e.)
(Intercept) -0.985 (0.357)
PiTfAaFn 0.003 (0.001)
Model: Type_Site ~ PiTfAaFn

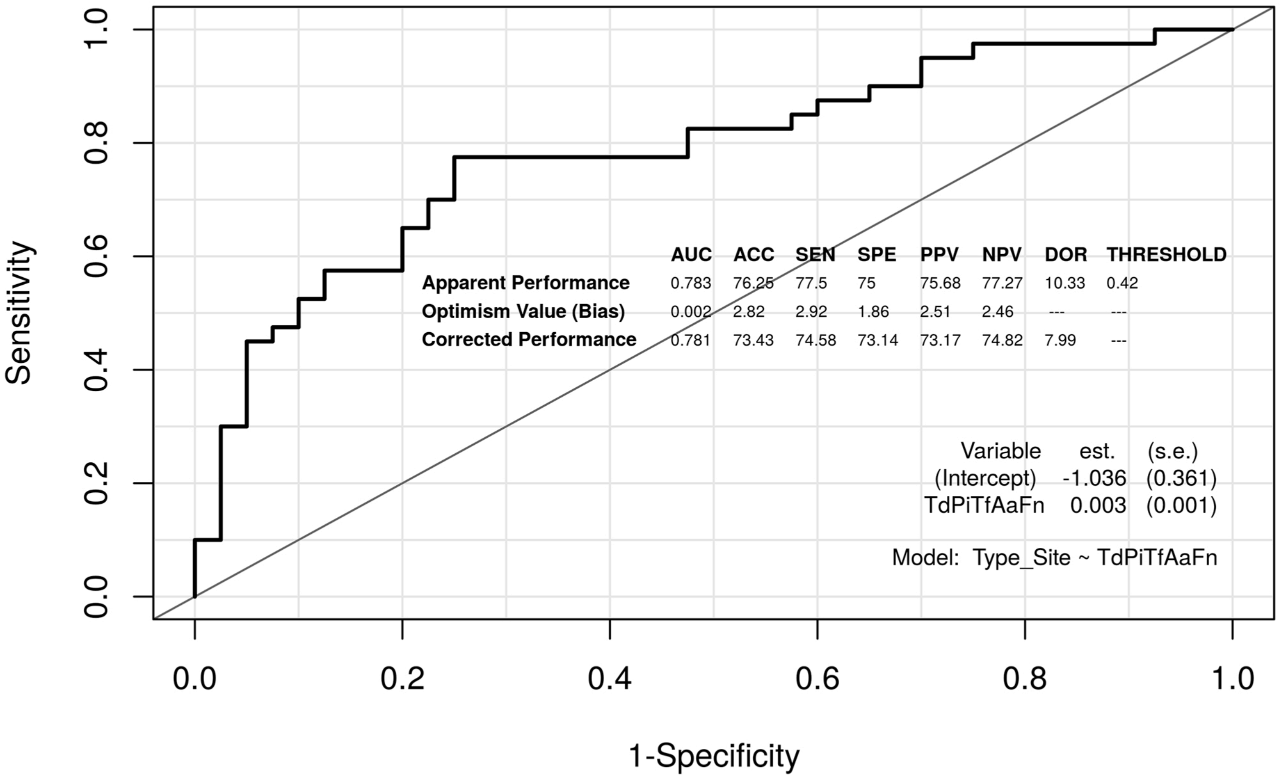
AUC ACC SEN SPE PPV NPV DOR THRESHOLD
Apparent Performance 0.783 76.25 77.5 75 75.68 77.27 10.33 0.42
Optimism Value (Bias) 0.002 2.82 2.92 1.86 2.51 2.46 --- ---
Corrected Performance 0.781 73.43 74.58 73.14 73.17 74.82 7.99 ---
Variable est. (s.e.)
(Intercept) -1.036 (0.361)
TdPiTfAaFn 0.003 (0.001)
Model: Type_Site ~ TdPiTfAaFn
Sensitivity
1-Specificity
0.0
0.2
0.4
0.6
0.8
1.0

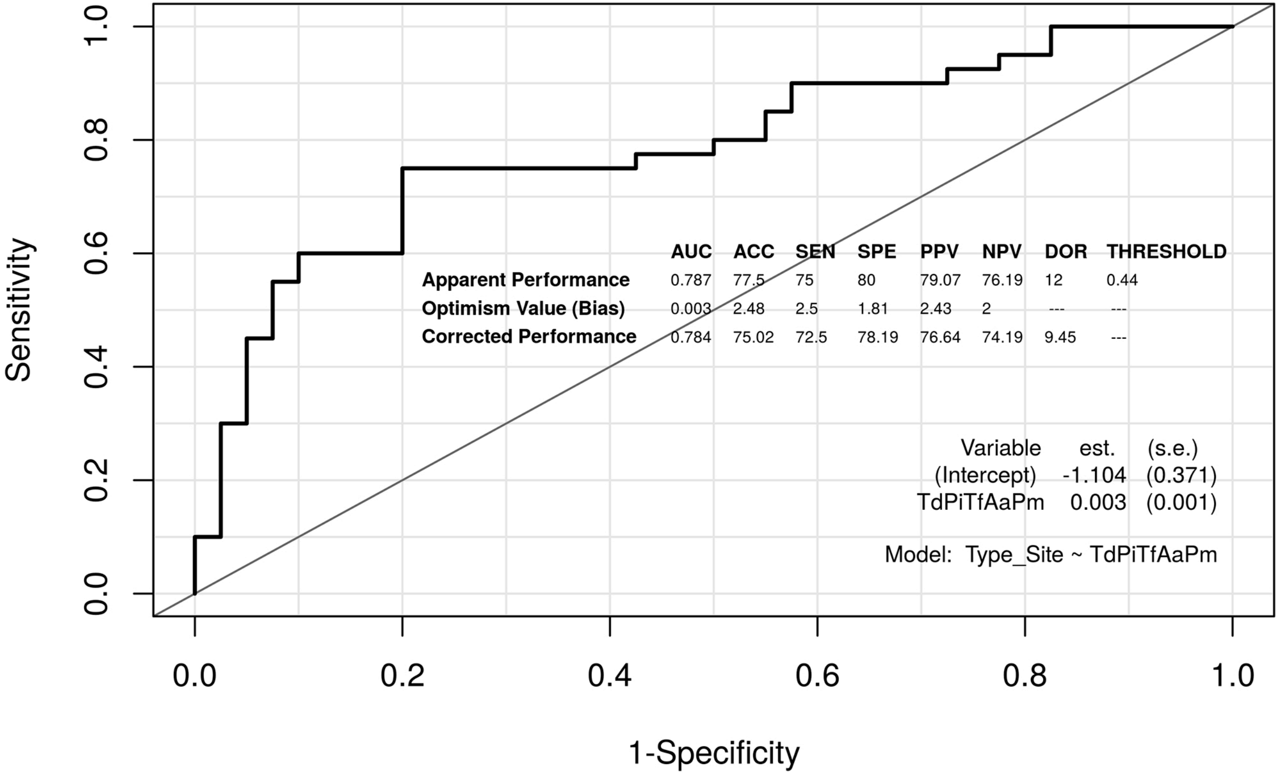

AUC ACC SEN SPE PPV NPV DOR THRESHOLD
Apparent Performance 0.787 77.5 75 80 79.07 76.19 12 0.44
Optimism Value (Bias) 0.003 2.48 2.5 1.81 2.43 2 --- ---
Corrected Performance 0.784 75.02 72.5 78.19 76.64 74.19 9.45 ---
Variable est. (s.e.)
(Intercept) -1.104 (0.371)
TdPiTfAaPm 0.003 (0.001)
Model: Type_Site ~ TdPiTfAaPm
Sensitivity
1-Specificity

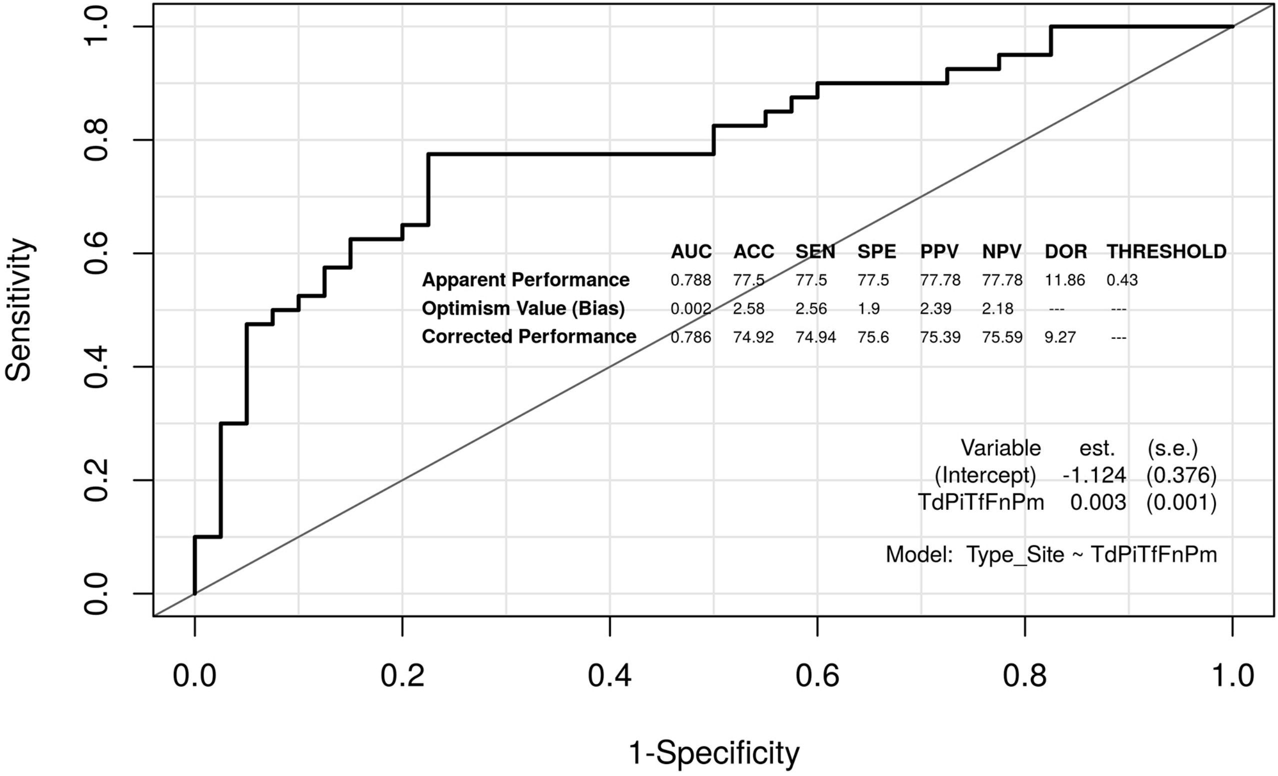

Sensitivity
1-Specificity
0.0
0.2
0.4
0.6
0.8
1.0
AUC ACC SEN SPE PPV NPV DOR THRESHOLD
Apparent Performance 0.788 77.5 77.5 77.5 77.78 77.78 11.86 0.43
Optimism Value (Bias) 0.002 2.58 2.56 1.9 2.39 2.18 --- ---
Corrected Performance 0.786 74.92 74.94 75.6 75.39 75.59 9.27 ---
Variable est. (s.e.)
(Intercept) -1.124 (0.376)
TdPiTfFnPm 0.003 (0.001)
Model: Type_Site ~ TdPiTfFnPm

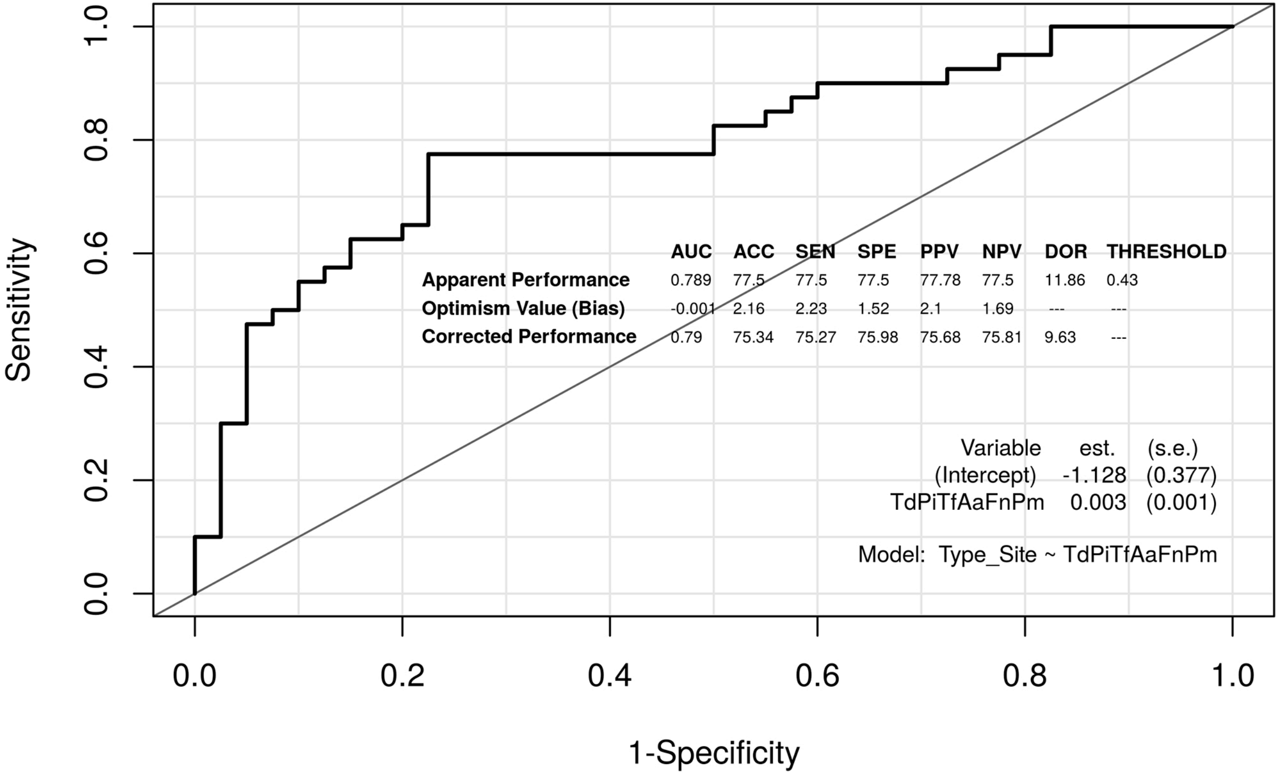

Sensitivity
1-Specificity
0.0
0.2
0.4
0.6
0.8
1.0
AUC ACC SEN SPE PPV NPV DOR THRESHOLD
Apparent Performance 0.789 77.5 77.5 77.5 77.78 77.5 11.86 0.43
Optimism Value (Bias) -0.001 2.16 2.23 1.52 2.1 1.69 --- ---
Corrected Performance 0.79 75.34 75.27 75.98 75.68 75.81 9.63 ---
Variable est. (s.e.)
(Intercept) -1.128 (0.377)
TdPiTfAaFnPm 0.003 (0.001)
Model: Type_Site ~ TdPiTfAaFnPm
