# Supplementary material for: Quantification by qPCR of Pathobionts in Chronic Periodontitis: Development of Predictive Models of Disease Severity at Site-Specific Level
Source: Front Microbiol. 2017 Aug 9;8:1443. doi: 10.3389/fmicb.2017.01443 (PMC5552702; doi:10.3389/fmicb.2017.01443)
Supplement: DATA SHEET S4 — (Figures S1–S8): Diagnostic nomograms derived from the cluster-based models with an AUC ≥0.76 and sensitivity and specificity values ≥75%. [file Data_Sheet_4.pdf]

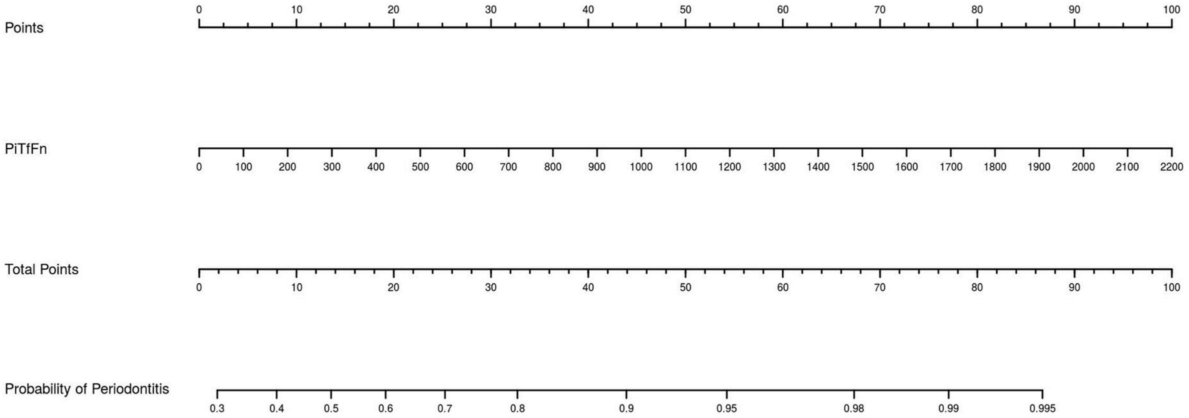
Points
0 10 20 30 40 50 60 70 80 90 100
PiTfFn
0 100 200 300 400 500 600 700 800 900 1000 1100 1200 1300 1400 1500 1600 1700 1800 1900 2000 2100 2200
Total Points
0 10 20 30 40 50 60 70 80 90 100
Probability of Periodontitis
0.3 0.4 0.5 0.6 0.7 0.8 0.9 0.95 0.98 0.99 0.995

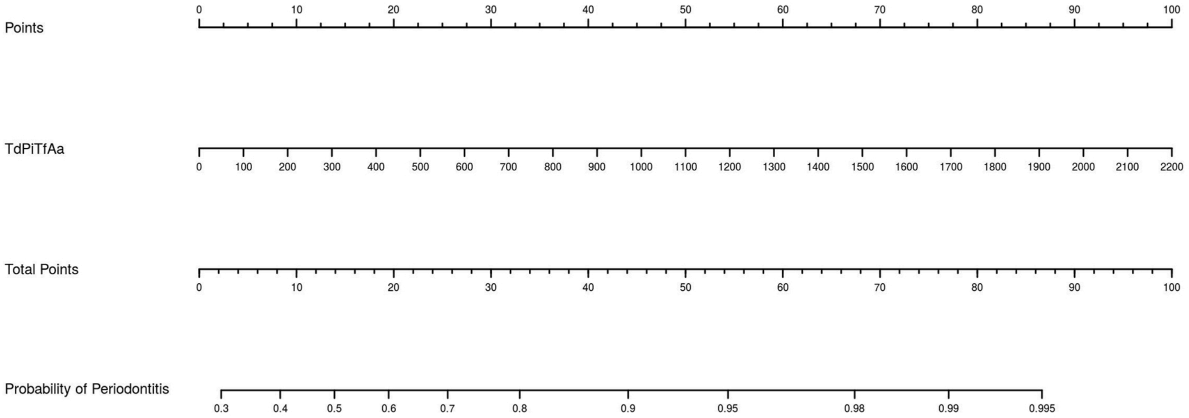
Points
0
10
20
30
40
50
60
70
80
90
100
TdPiTfAa
0
100
200
300
400
500
600
700
800
900
1000
1100
1200
1300
1400
1500
1600
1700
1800
1900
2000
2100
2200
Total Points
0
10
20
30
40
50
60
70
80
90
100
Probability of Periodontitis
0.3
0.4
0.5
0.6
0.7
0.8
0.9
0.95
0.98
0.99
0.995

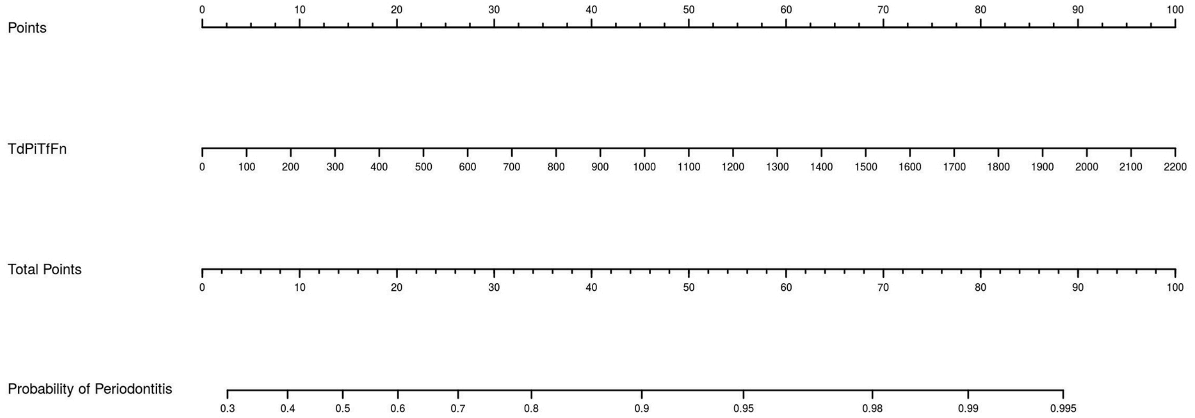
Points
0 10 20 30 40 50 60 70 80 90 100
TdPiTfFn
0 100 200 300 400 500 600 700 800 900 1000 1100 1200 1300 1400 1500 1600 1700 1800 1900 2000 2100 2200
Total Points
0 10 20 30 40 50 60 70 80 90 100
Probability of Periodontitis
0.3 0.4 0.5 0.6 0.7 0.8 0.9 0.95 0.98 0.99 0.995

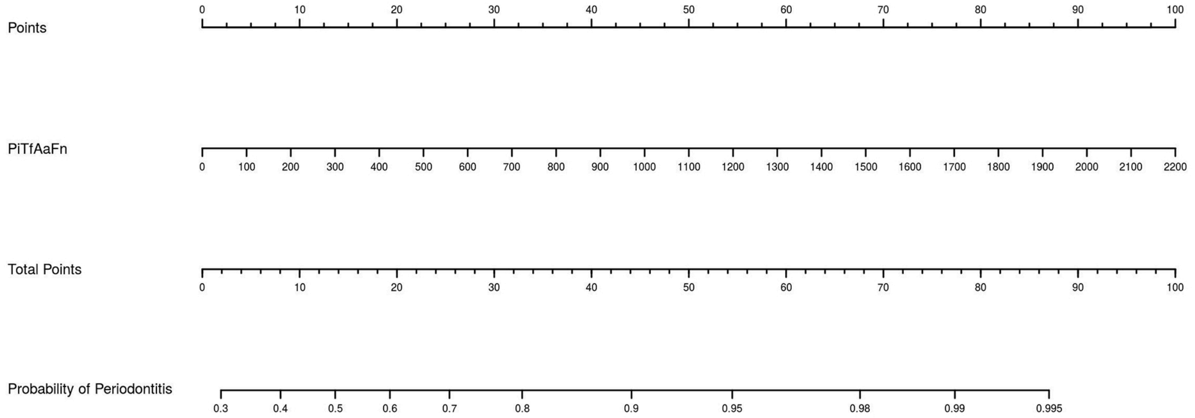
Points
0 10 20 30 40 50 60 70 80 90 100
PiTfAaFn
0 100 200 300 400 500 600 700 800 900 1000 1100 1200 1300 1400 1500 1600 1700 1800 1900 2000 2100 2200
Total Points
0 10 20 30 40 50 60 70 80 90 100
Probability of Periodontitis
0.3 0.4 0.5 0.6 0.7 0.8 0.9 0.95 0.98 0.99 0.995

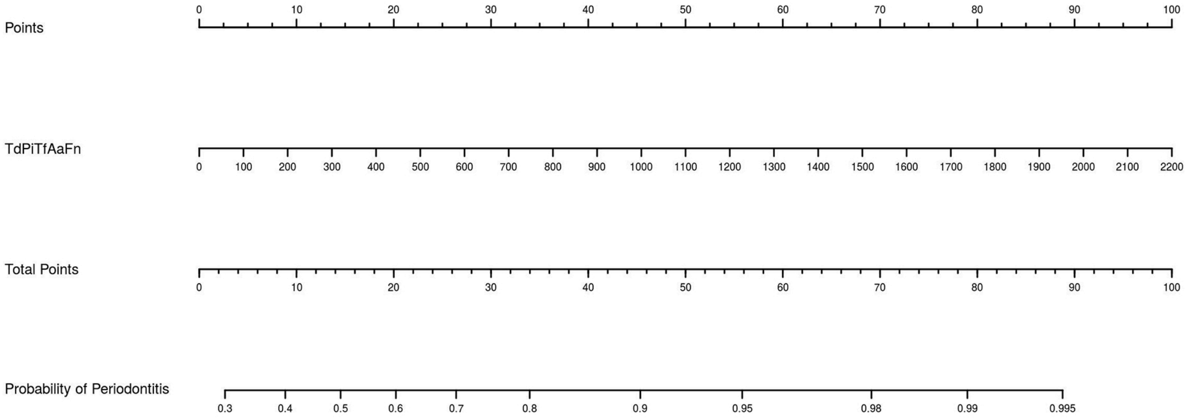
Points
0 10 20 30 40 50 60 70 80 90 100
TdPiTfAaFn
0 100 200 300 400 500 600 700 800 900 1000 1100 1200 1300 1400 1500 1600 1700 1800 1900 2000 2100 2200
Total Points
0 10 20 30 40 50 60 70 80 90 100
Probability of Periodontitis
0.3 0.4 0.5 0.6 0.7 0.8 0.9 0.95 0.98 0.99 0.995

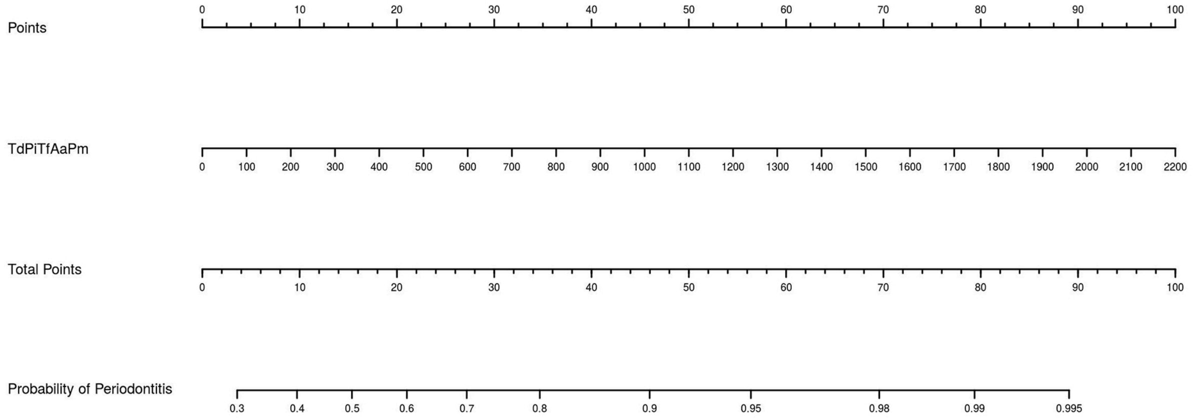

Points
0 10 20 30 40 50 60 70 80 90 100
TdPiTfAaPm
0 100 200 300 400 500 600 700 800 900 1000 1100 1200 1300 1400 1500 1600 1700 1800 1900 2000 2100 2200
Total Points
0 10 20 30 40 50 60 70 80 90 100
Probability of Periodontitis
0.3 0.4 0.5 0.6 0.7 0.8 0.9 0.95 0.98 0.99 0.995

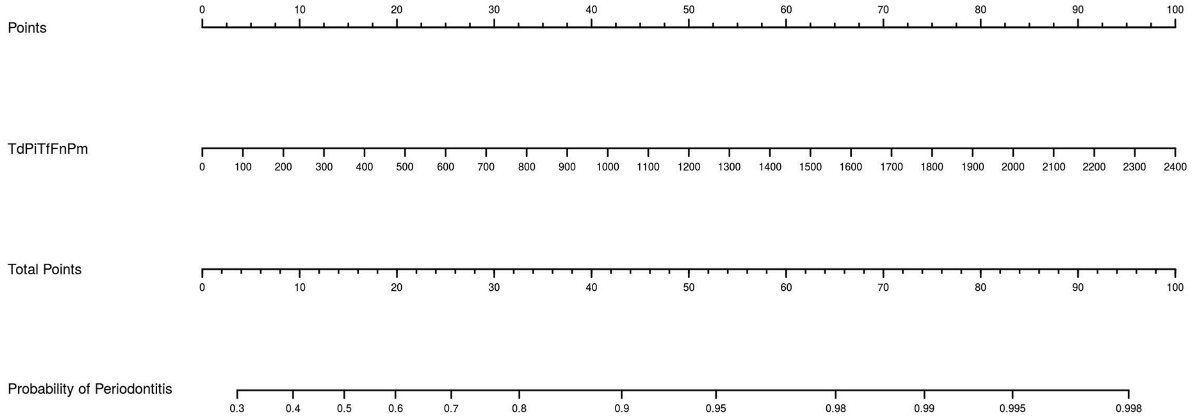
Points
0 10 20 30 40 50 60 70 80 90 100
TdPiTfFnPm
0 100 200 300 400 500 600 700 800 900 1000 1100 1200 1300 1400 1500 1600 1700 1800 1900 2000 2100 2200 2300 2400
Total Points
0 10 20 30 40 50 60 70 80 90 100
Probability of Periodontitis
0.3 0.4 0.5 0.6 0.7 0.8 0.9 0.95 0.98 0.99 0.995 0.998

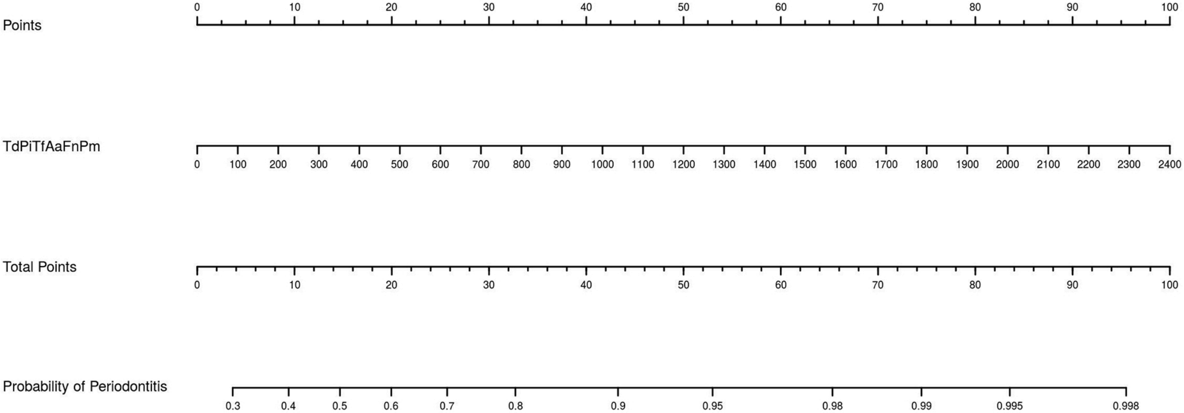
Points
0 10 20 30 40 50 60 70 80 90 100
TdPiTfAaFnPm
0 100 200 300 400 500 600 700 800 900 1000 1100 1200 1300 1400 1500 1600 1700 1800 1900 2000 2100 2200 2300 2400
Total Points
0 10 20 30 40 50 60 70 80 90 100
Probability of Periodontitis
0.3 0.4 0.5 0.6 0.7 0.8 0.9 0.95 0.98 0.99 0.995 0.998
